# Expanding kinetoplastid genome annotation through protein structure comparison

**DOI:** 10.1371/journal.ppat.1013120

**Published:** 2025-04-21

**Authors:** Juan Manuel Trinidad-Barnech, José Sotelo-Silveira, Darío Fernández Do Porto, Pablo Smircich

**Affiliations:** 1 Laboratorio de Bioinformática, Departamento de Genómica, Instituto de Investigaciones Biológicas Clemente Estable, MEC, Montevideo, Uruguay; 2 Laboratorio de Genómica Evolutiva, Sección Biomatemática, Facultad de Ciencias, Universidad de la República, Montevideo, Uruguay; 3 Departamento de Genómica, Instituto de Investigaciones Biológicas Clemente Estable, MEC, Montevideo, Uruguay; 4 Sección Biología Celular, Facultad de Ciencias, Universidad de la República, Montevideo, Uruguay; 5 Instituto de Cálculo, Facultad de Ciencias Exactas y Naturales, Universidad de Buenos Aires, Buenos Aires, Argentina; 6 Departamento de Química Biológica, Facultad de Ciencias Exactas y Naturales, Universidad de Buenos Aires, Buenos Aires, Argentina; 7 Sección Genómica Funcional, Facultad de Ciencias, Universidad de la República, Montevideo, Uruguay; University of Texas Southwestern Medical School, UNITED STATES OF AMERICA

## Abstract

Kinetoplastids belong to the Discoba supergroup, an early divergent eukaryotic clade. Although the amount of genomic information on these parasites has grown substantially, assigning gene functions through traditional sequence-based homology methods remains challenging. Recently, significant advancements have been made in *in-silico* protein structure prediction and algorithms for rapid and precise large-scale protein structure comparisons. In this work, we developed a protein structure-based homology search pipeline (ASC, Annotation by Structural Comparisons) and applied it to transfer biological information to all kinetoplastid proteins available in TriTrypDB, the reference database for this lineage. Our pipeline enabled the assignment of structural similarity to a substantial portion of kinetoplastid proteins, improving current knowledge through annotation transfer. Additionally, we identified structural homologs for representatives of 6,700 uncharacterized proteins across 33 kinetoplastid species, proteins that could not be annotated using existing sequence-based tools and databases. As a result, this approach allowed us to infer potential biological information for a considerable number of kinetoplastid proteins. Among these, we identified structural homologs to ubiquitous eukaryotic proteins that are challenging to detect in kinetoplastid genomes through standard genome annotation pipelines. The results (KASC, Kinetoplastid Annotation by Structural Comparison) are openly accessible to the community at kasc.fcien.edu.uy through a user-friendly, gene-by-gene interface that enables visual inspection of the data.

## Summary

Kinetoplastids are a group of parasites that cause severe diseases in the poorest regions of the world. Despite the increasing amount of genomic information available on these parasites, predicting the function of many of their genes using traditional methods has been difficult. Recently, there have been significant advancements in predicting protein structures and comparing them on a large scale. In this study, we developed a method called ASC (Annotation by Structural Comparisons) to bioinformatically infer putative functions for all kinetoplastid genes listed in the TriTrypDB database. Our strategy allowed us to transfer biological information to 6,700 proteins in kinetoplastids currently named hypothetical in the database which could only be achieved using protein structural information. Also, the results significantly enhance the currently available annotation in the database for a significant proportion of the kinetoplastid proteins. Among these, we identified homologs of important proteins found in most eukaryotes. This information (KASC, Kinetoplastid Annotation by Structural Comparison) is accessible at kasc.fcien.edu.uy.

## Introduction

Kinetoplastids are flagellated protozoan parasites belonging to the early branching Discoba supergroup [1]. While the amount of genomic information on these parasites has grown substantially, a significant challenge the community faces is the high percentage of genes lacking functional annotation, impeding comprehensive interpretation of genome-wide studies [2]. This deficiency in functional assignment is attributed not only to species-specific genes but also to the kinetoplastids divergence within the eukaryotic lineage [1].

Functional annotation of proteins is crucial for understanding cellular biology at the molecular level. However, rapid increases in gene sequences driven by high-throughput technologies challenge traditional methods relying on extensive manual curation. Even though this kind of effort is being performed for some genomes such as *Plasmodium falciparum* or haloarchaeal [3–5], automated annotation methods are increasingly necessary to overcome this bottleneck. Historically, functional annotation strategies depend mainly on the transfer of information from experimentally characterized protein sequences by homology inference, conserved functional domains (mostly based on Hidden Markov Models), or supervised learning approaches (Position-Specific Score Matrices) [6–9]. Despite the success of sequence-based homology inference, detecting distant evolutionary relationships remains challenging for these approaches [10].

Since protein structure is commonly more conserved than the amino acid sequence, structural alignment methods are especially relevant in phylogenetically distant organisms, offering greater sensitivity to identify homology [10,11]. However, the large-scale use of this approach was always limited by the availability of experimentally determined protein structure data, the difficulty of predicting protein structure from only its amino acid sequence, and the speed of structural comparison methods [12].

Recently, significant advancements have been made in the field of *in silico* structure prediction, solving this problem for many proteins and opening new possibilities in structural biology and bioinformatics [12–15]. Algorithms such as AlphaFold [13], RoseTTAFold [15], and ESMFold [14] now allow the prediction of three-dimensional protein structures based solely on amino acid sequences, achieving accuracy competitive with experimental results. The availability of comprehensive protein structures significantly enhances our ability to identify structurally similar proteins. This progress is driven by algorithms that enable rapid and precise large-scale protein structure comparisons, such as Foldseek [12]. These algorithms facilitate efficient protein structure searches in extensive databases like AlphaFold Database (AFDB) [16] and the ESM Metagenomic Atlas [14] allowing the development of new strategies to search for distant homology. Analogous to sequence-based reciprocal best hits (e.g., BLAST), structural reciprocal best hits (SRBH) are being used to improve orthology/homology inference, functional annotation, and evolutionary analysis [17]. SRBH has shown potential in detecting novel homologs between distant reference organisms within Opisthokonta [17].

While reciprocal best hit (RBH) is commonly used to assign orthologs and serve as a proxy for functional conservation, distinguishing orthologs from paralogs over long evolutionary distances remains a significant challenge due to gene duplication and loss events [18]. Furthermore, the relationship between structural and functional similarity is complex, as there are examples of proteins with similar structures performing different functions [18]. A comprehensive assessment of the strengths and weaknesses associated with employing these tools is ongoing. Recent analysis suggests that BLAST offers precise homolog identification with stringent e-values, hidden Markov models provide higher sensitivity but lower specificity, and structure comparison reaches a balance between these approaches [10].

In this work, we propose an approach based on SRBH to detect distant homologs and transfer putative biological information to the genomes of the kinetoplastid group. To achieve this aim, we developed a user-friendly tool and applied it to kinetoplastids genomes. Our results demonstrate the effectiveness of this strategy, producing concordant annotations for proteins with known functions in the database. Moreover, we assigned putative functions to thousands of genes named as hypothetical in the reference kinetoplastid database. While gene names may not necessarily indicate a lack of biological information, the propagation of new annotations depends on several factors beyond the scope of this study. However, we confirmed that most of these genes lack any annotation (e.g., no associated GO terms or protein domains). Additionally, we showed that standard sequence-based annotation pipelines, such as eggNOG [19] and InterProScan [20] are not capable of annotating a significant proportion of these genes. The tool (ASC, Annotation by Structural Comparison) and the corresponding results for kinetoplastid genomes (KASC, Kinetoplastid Annotation by Structural Comparison) are openly available to the community. Additionally, bulk downloads for the TriTryps proteins are available at https://zenodo.org/records/14397343. The complete dataset is available upon request.

## Results

### ASC (Annotation by Structural Comparisons) pipeline description

The entire workflow was implemented in the Snakemake workflow management system [21], a popular tool for automating bioinformatics workflows. The execution script and the required dependencies are available at https://github.com/JuanTrinidad/ASC. A configuration file located in the “config” directory allows for the adjustment of all parameters mentioned in the methods section (as well as others configurable according to the documentation of each software). This pipeline was developed not only to run the analyses presented in this manuscript but also to provide a tool that users can easily implement for their organism of interest.

**Fig 1:**
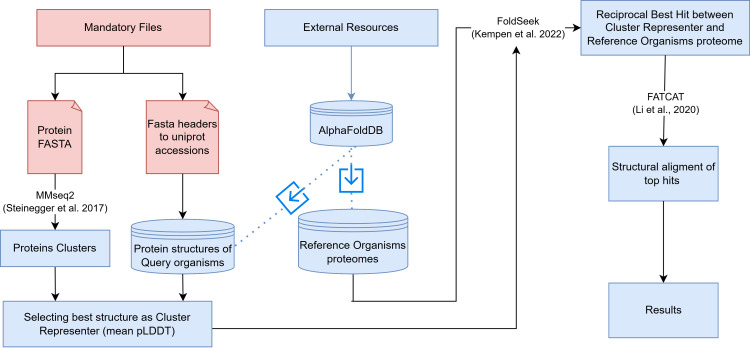
ASC pipeline workflow. Workflow outlining the process for homology search of proteins using protein structural data. The pipeline begins with two mandatory files, a FASTA file with protein sequences and a TSV file linking FASTA headers with the corresponding UniProt accessions. MMseq2 is employed to generate protein clusters. All protein sequences with available structures in AFDB will be downloaded, and the best structural representative for each cluster will be selected based on mean pLDDT scores. Foldseek is used to identify SRBHs between cluster representatives and reference organism proteomes. Top hits undergo structural alignment using FATCAT, and TM-score filtering leading to the results.

The workflow requires two files: a fasta file containing the protein sequences to be annotated and a TSV (Tab-Separated Values) file linking the fasta headers with a UniProt accession. The workflow initiates by clustering the protein sequences to be analyzed using MMseq2 [22] (see Materials and Methods) and setting the minimum number of proteins per cluster to be retained. Once the sequences are clustered, the clusters are filtered by the number of sequences defined by the user. Using the second mandatory TSV file, the pipeline downloads all available structures for the sequences within the clusters via FTP from AFDB. Each protein structure will be compared with the sequences in the fasta file to prevent inconsistencies that might exist between sequences in cases where sequences are obtained from different databases (see Materials and Methods). Since clusters can contain multiple structures, we choose the best structure prediction for each cluster based on the average pLDDT. These initial steps result in the selection of a representative protein for each cluster. This approach has a limitation because structural homology searches using non-representative sequences may differ in specificity and accuracy compared to searches using the selected representative structure. Despite this limitation, our strategy improves pipeline efficiency by reducing the number of structural comparisons performed and minimizing redundant results. Furthermore, as many organisms are not currently included in the AFDB, this approach enables the transfer of information to other proteins within the clusters that lack structural predictions. The pipeline then creates a database for the Foldseek [12] program using these representative structures (query database). The remaining clusters without available protein structures are excluded from the analysis.  In parallel, the Snakemake script downloads all available reference organisms from AFDB and creates an independent database for each organism. These databases are used as targets.  Using Foldseek RBH (equivalent to SRBH) comparisons are performed between the query database and each target database independently (generating one comparison of the query per model organism). For each query, the pipeline selects the top number of SRBH as specified by the user (sorted by Foldseek e-value) and performs a flexible structural alignment between the corresponding PDB files using the FATCAT algorithm [23]. FATCAT is a structure aligner that allows for flexibility, an important aspect since AlphaFold structures often have different domain topologies/orientations [24]. For each protein structure comparison, the TM-scores used to filter significant similarities are obtained from the FATCAT results. [25] (Fig 1). The final result consists of a table with all SRBH results from Foldseek, results from FACTCAT, and target structure annotation from UniProt [26].

### Structural homology search of kinetoplastid proteins

The development of this pipeline enabled us to analyze 657,192 kinetoplastid protein sequences from all genomes available in the public kinetoplastid database TriTrypDB [27]. Proteins were clustered, and groups were filtered by size selecting clusters of at least 10 sequences. This criterion was used to avoid further analysis of incorrect gene predictions or genes lacking homology across kinetoplastid genomes. For this study, we used the gene description field in the reference database TriTrypDB to initially identify a set of proteins named as hypothetical. We then tested whether structural homology searches could be used to assign them putative functional annotations. It is important to note that this set may also include proteins with experimental evidence of expression (e.g., revealed by RNA-seq), evidence from the literature that was not integrated in the database or cases where available information is not reflected in the gene name. To explore these possibilities, we tested whether members of clusters composed solely of proteins named hypothetical, where we identified structural homologs, could be annotated using existing methods and databases, including eggNOG and InterProScan (see below). We also identified those that had further information in the database, regardless of their gene name. If a gene received a hit from any of the strategies all genes within its cluster were excluded.

The initial clustering and selection steps discarded 117,582 hypothetical proteins and 61,198 annotated proteins, representing genes with insufficient genomes to meet the minimum number of homologs, such as species-specific genes, pseudogenes, highly variable sequences, or artifacts generated during genome annotation. The significant loss of genes during these steps underscores the challenges inherent in genome annotation for kinetoplastids. Additionally, genome annotation methods may introduce artifacts through misprediction of coding regions, particularly in species with fragmented or incomplete genome assemblies [28]. The stringent clustering threshold helps to ensure robust downstream analyses but inevitably excludes real gene predictions. These losses reflect the current limitations of homology-based approaches in capturing the full diversity of kinetoplastid genes.

**Fig 2:**
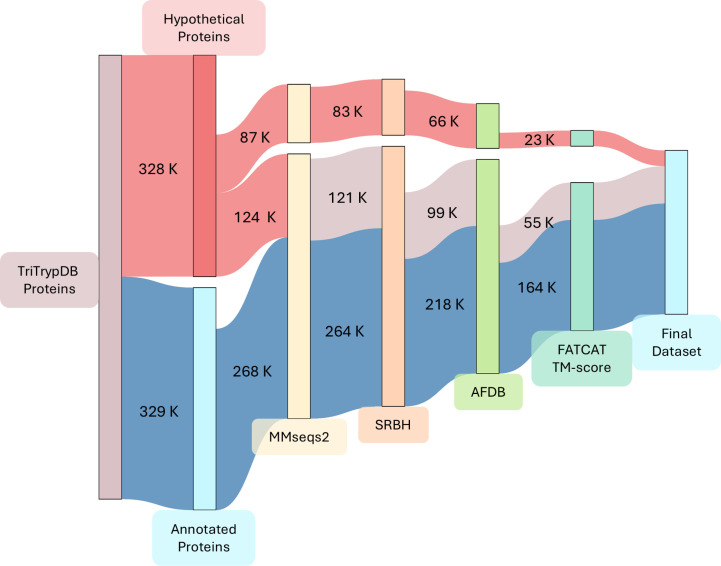
The number of protein sequences retained throughout the main steps of the pipeline. Sankey diagram visualizing the number of genes retained after each main step. The nodes in the diagram, each represented by a specific color, denote the pipeline’s stages, while the links between nodes indicate the number of genes (in thousands) transitioning from one step to the next. The color coding distinguishes between hypothetical and annotated genes as per TriTrypDB: red represents genes named hypothetical, blue represents annotated genes, and light red represents hypothetical genes with sequence homology to annotated genes. Through protein sequence clustering (MMseqs2), structure retrieval (AFDB), Foldseek reciprocal best hit (SRBH), and structural alignment (FATCAT & TM-score), the workflow filters to the Final Dataset.

This process resulted in 14,778 clusters encompassing 478,612 sequences, of which 210,201 (44%) were labeled as hypothetical proteins. Among these, 3,565 clusters, comprising 86,679 sequences, consisted solely of hypothetical proteins (termed “Dark Clusters” [29]). The remaining 123,522 proteins named hypothetical (representing 59% of the total) were grouped with currently annotated protein sequences. So, due to our stringent clustering parameters we were able to transfer annotation to a significant number of these proteins based solely on sequence homology [29,30]. Notably, 90% of our clusters correspond to a single ortholog group defined by OrthoMCL [31] in TriTrypDB, providing additional validation for the annotation transfer. Considering both strategies, transferring annotations within these groups appears to be a justified approach. Of the 14,778 retained clusters, 14,267 had protein structures available in AFDB (which includes 20 Leishmania, 20 Trypanosoma, 2 Leptomonas, *Bodo saltans*, *Porcisia hertigi* proteomes), which served as our query database for subsequent pipeline steps. The remaining 511 clusters, lacking available protein structures, were excluded from the analysis. For the clusters where a representative structure could be obtained, we performed the SRBH step against reference organisms (excluding trypanosomatids and leishmanias) retaining 11,753 protein clusters, including 2,689 Dark Clusters. This collection will be referred to as the “Raw Dataset”. We selected the top five SRBH for each query, conducted structural alignments using FATCAT against the top targets reported by Foldseek, and extracted TM-score values. Using a stringent TM-score threshold of 0.5 for protein homology [32], we filtered the results (both TM-score Chain 1 and TM-score Chain 2 > 0.5), creating our “Final Dataset”. Although the TM‐score threshold of 0.5 was originally defined for single-domain proteins [27], it has been used as reference in multi-domain proteins alignments as well [32,33]. It is worth noting that the TM-score considers the alignment length between hits. Our chosen parameters prioritize full protein alignments, excluding partial alignments that might arise from similar subdomains. The Final Dataset, derived from our rigorous pipeline steps and TM-score filtering, resulted in 7,486 clusters, including 942 Dark Clusters comprising 23,290 protein sequences (Fig 2). It is important to note that these clusters were defined as “Dark” based on the current TriTrypDB gene names. Therefore, the number of Dark Clusters and the improvements achievable through structure-based homology searches depend on this specific field of each gene entry and does not account for additional information that may not be incorporated into the protein name in the database. This prompted us to investigate whether any additional information was available for these proteins in the database. We found that 3,944 proteins indeed contain annotations, in the form of assigned GO terms, protein domain identifiers, etc. Moreover, with the growing number of annotated organisms and improvements in functional annotation methods, it is possible that some of these proteins could now have identifiable sequence homologs or domains if reanalyzed. So, we assessed whether the annotation of these proteins could be improved using current sequence-based approaches. To this end, we ran eggNOG and InterProScan, on the approximately 23,000 proteins from Dark Clusters for which we identified structural homologs. This analysis revealed that 1,700 proteins could be annotated using eggNOG and for approximately 4,000 a domain could be identified using InterProScan. Ultimately, integrating information from all approaches and transferring it within cluster members reinforces that a significant proportion of the 23,000 proteins in the Dark Clusters (approximately 6,700) could only be assigned putative annotation through structural comparisons.

Proteins belonging to these clusters are particularly interesting because they appear in multiple kinetoplastid genomes or have multiple copies within a single genome and lack sequence homology with genes annotated in TriTrypDB. Notable examples are discussed in the last section of the article.

When putative annotation is transferred to kinetoplastid proteins from structurally similar genes from model organisms, we observed that, as expected, GO terms associated with these new annotations span a diverse range of functional categories. Notably, several categories such as membrane-associated were overrepresented compared to their frequency in the previously annotated terms (S1 Fig). It is important to emphasize that, irrespective of whether a function can be transferred to these clusters based on information from the reference organisms, finding a structural similar protein might indicate that these genes are conserved among highly divergent organisms and thus are likely functionally relevant. Moreover, the impact of convergent evolution on our findings is expected to be minimal, as 97.5% of the SRBH results in our Final Dataset have e-values below 10 ⁻^²^. Previous studies have demonstrated that e-values below this threshold significantly reduce the likelihood of convergent evolution [29].

The results are accessible through a browsable web server at kasc.fcien.edu.uy. The default tab allows users to query a GeneID, displaying TriTrypDB and UniProt annotations, the top five structural comparison results, and a visualization of the structural alignment of the queried protein against a selected organism. Since the results are not manually curated, the hits are color-coded based on the TM-score to indicate the reliability of the predictions. The secondary tab provides information on the corresponding cluster that the ID belongs to and a detailed description of the structural alignment.

### Validation of annotation transfer

**Fig 3:**
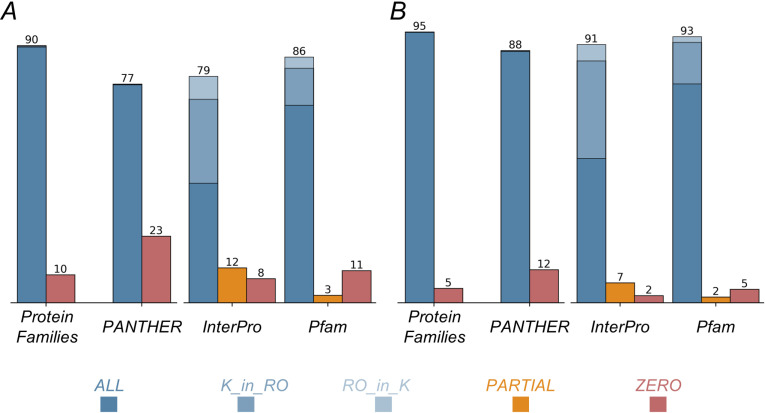
Evaluation of SRBH annotations before and after filtering. Comparison of SRBH annotation results for query and target proteins using four databases: Protein Families, PANTHER, InterPro, and Pfam. The left panel (A) displays the annotation results before applying a filtering process, while the right panel (B) shows the results after filtering (Final Dataset). Each bar represents the percentage of annotations categorized as ALL (blue), K_in_RO (light blue), RO_in_K (lighter blue), PARTIAL (orange), and ZERO (red). The numbers at the top of each bar indicate the total percentage of the column rounded.

To evaluate the accuracy of annotation transfer between the reference organism and the kinetoplastid SRBHs, we examined whether the transferred annotations assigned similar functions to already annotated kinetoplastid proteins. We assessed consistency in two datasets: the Raw Dataset (based solely on Foldseek SRBHs) (Fig 3A) and the Final Dataset (incorporating Foldseek SRBHs with FATCAT-TM-score filtering) (Fig 3B).

For each pair of SRBHs, we retrieved protein annotations from UniProt using the Protein Families, PANTHER, InterPro, and Pfam databases for both kinetoplastid and reference organism proteins. The level of agreement between annotations was classified into five possible categories. ALL: Identical annotations between kinetoplastid (“K”) and reference organism (“RO”) proteins; K_in_RO: Annotations in K were a subset of those in RO; RO_in_K: Annotations in RO were a subset of those in K; PARTIAL: K and RO shared some, but not all, annotations; ZERO: No overlap between K and RO annotations.  For the Protein Families and PANTHER databases, only the ALL and ZERO categories apply, as these databases provide a single level of annotation that either matches or does not match. In contrast, for InterPro and Pfam databases, which can include multiple independent domains, the additional categories (K_in_RO, RO_in_K, and PARTIAL) are particularly relevant.

Our results from the Protein Families and PANTHER databases show that our annotation is highly consistent with protein family-level annotations. The data shows an enhancement in annotation precision after filtering from Raw Dataset to Final Dataset, as seen by the increased number of exact matches (ALL category) and the reduction of non-overlapping annotations considered incorrect assignments (ZERO category) (Fig 3A and 3B). In the first case, we obtain a 90% success rate in Raw Dataset, increasing to 95% in the Final Dataset. These results are similar to those of the PANTHER database at the superfamily level where we are at 77% accuracy, increasing to 88% in the Final Dataset (Fig 3A and 3B). Therefore, our results show that proteins with an existing family assignment exhibit a high level of concordance with the SRBH classification in the model organism. So, when transferring annotation we are facing a reliable family prediction. This is comparable to the results obtained by previous studies in organisms even more closely related to each other (inside the Opisthokonta clade) [17].

At the domain level, we also have an increase in the ALL and a decrease in the ZERO and PARTIAL categories from Raw Dataset to Final dataset. Using the InterPro and Pfam databases, within the ALL category, we distinguish between exact matches (dark blue) and matches where the domains of one protein are a subset of the other (light blues, see Methods). As shown in Fig 3, we observe 79% and 86% coincidence in the Raw Dataset, rising to 91% and 93% in the Final Dataset.  It is worth noting that the light blue data (cases where one set is completely included in the other) is mostly composed of the K_in_RO category meaning that the kinetoplastid domains correspond to a subset of the ones in the model organisms. So, applying our pipeline and transferring annotation from structural homologs would result in a considerable advance for kinetoplastid proteins even for the ones that already have domains annotated. Considering this in our Final Dataset, annotation transfer added new annotation terms to 34% of the clusters in InterPro and 15% in Pfam (K_in_RO).

This improvement is reflected in the reanalysis of previously published data, where annotation transfer from the reference organism increased the depth of GO categories overrepresented in differentially expressed gene lists [34,35] (S2 Fig). Interestingly, manual analysis of these gene lists provided biological insights into proteins currently named hypothetical in TriTrypDB. For example, the *T. brucei* protein *Tb927.9.6380* has an SRBH with metabolite/drug transporters. Interestingly, two independent user comments in TriTrypDB associate this gene with drug resistance mechanisms. Furthermore, the functional inference is supported by consistent annotations in members of the MMseq2 clusters we constructed (Major Facilitator Superfamily, known transporter proteins linked to drug transport and resistance mechanisms), as well as OrthoMCL groups in TriTrypDB.

In the opposite case, where an annotation in reference organisms is not observed in the kinetoplastid protein (RO_in_K), it is more difficult to affirm that this is due to a lack of annotation. However, the percentage of these cases is low. Also, the PARTIAL and ZERO matches are low, as both added do not reach 10% in the Final Dataset.

All comparisons between the annotation databases in Raw Dataset versus Final Dataset were assessed using the Chi-squared test, and all showed significant differences (p-value < 1e-10).

### TrypTag validation

TrypTag is a genome-wide experimental database that details protein locations within the *T. brucei* parasite. Researchers used fluorescent tags and microscopy to determine protein locations, and each tagged protein was manually annotated for its subcellular location using a standardized ontology [36]. This database is a highly accurate reference for our approach, enabling us to validate our tentative transferred annotations against a gold standard. To achieve this, we assessed the accuracy of transferring subcellular localization information from structural homologs in model organisms to kinetoplastid proteins, using the TrypTag-reported localization as a reference. As a control, we also evaluated the consistency of the current subcellular localization annotations for kinetoplastid proteins against the TrypTag data.  To enhance the reliability of our analysis, we removed non-confident entries and selected ubiquitous GO cellular components (CC) from TrypTag (see Methods) and used the GO CC annotation from our Final Dataset to compare.

**Fig 4:**
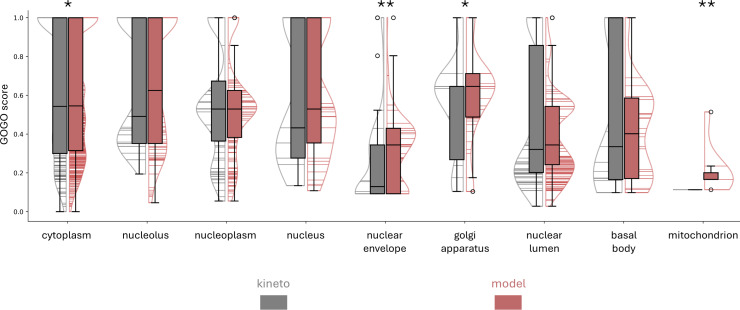
Comparison of GO cellular component semantic similarity scores for kinetoplastid and reference organism annotations. Distribution of GO term semantic similarity scores (GOGO scores) for two categories: kinetoplastid (grey) and reference organism (red). Each violin plot displays the range and distribution of scores for ubiquitous cellular components, including cytoplasm, nucleus, nuclear lumen, nuclear envelope, nucleolus, basal body, golgi apparatus, and mitochondrion. The boxplot within each violin plot represents the interquartile range (IQR) and the white lines indicate the median values. * p-value < 0.05, ** p-value < 0.01.

When analyzing an SRBH pair, various scenarios arise regarding the presence of a GO CC annotation: it may only be present for the kinetoplastid protein, only present for the reference organism protein, present for both, or absent for both. To address this, we independently compared the GO CC annotations of the kinetoplastid and reference organism proteins (obtained from UniProt) with the annotations assigned by TrypTag for each SRBH pair, whenever possible. We assessed the semantic similarity of the GO terms using GOGO [37]. Fig 4 shows the distribution of semantic similarity values of GO terms for each comparison. The grey violin plots represent the distribution of GOGO scores by comparing the annotation of kinetoplastid genes (current Uniprot annotation) with the annotations assigned by TrypTag, which serves as our reference. The red violin plots represent the results from comparing the annotation data of reference organisms with the annotations of TrypTag, which approximates the confidence level we can obtain with our annotation strategy for hypothetical proteins.

Using the Mann-Whitney-U test to compare the means, we did not observe significant differences for most categories, meaning that the current annotation is equally coherent with TrypTag as our analysis. We observe a trend for cytoplasm, nuclear envelope, and Golgi apparatus, indicating that SRBH strategy annotates subcellular localizations more in line with the TrypTag experimental results. In conclusion, our annotation is at least as good as the currently accepted methods when compared against TrypTag, and it contributes new information for currently uncharacterized proteins.

### Case study

After validating our approach, we sought biologically relevant examples within our results, focusing on Dark Clusters and Annotated Clusters lacking precise annotations. To refine our search and identify truly Dark Clusters using various databases, we incorporated functional annotation data from UniProt, specifically the Product Description, in addition to TriTrypDB.

**Fig 5:**
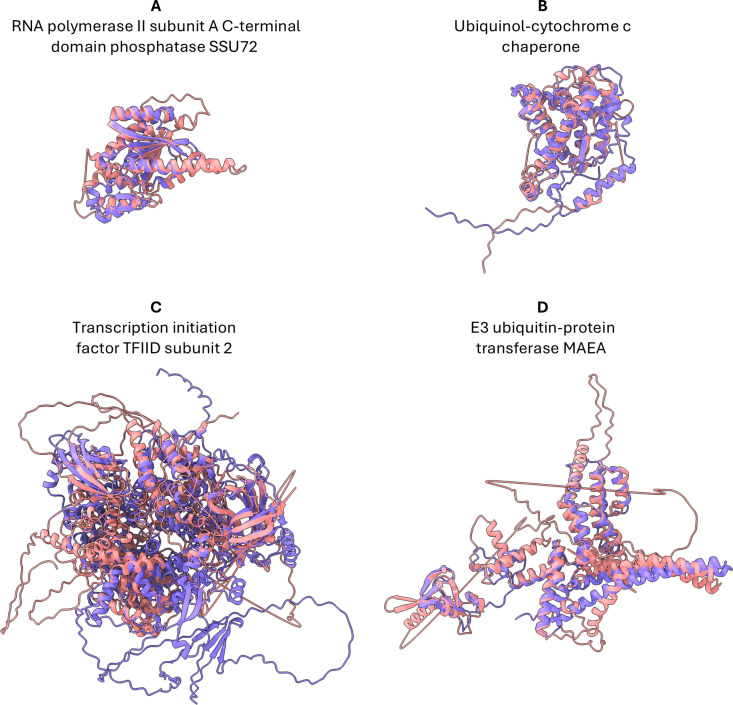
Structural alignment of ubiquitous eukaryotic proteins with kinetoplastid homologs identified through our approach. The target protein (reference species) is shown in purple, and the query protein (kinetoplastids) is shown in pink. In most cases, there are SRBH with homologs of this protein in different organisms; we chose one as an example. (A) RNA polymerase II subunit A C-terminal domain phosphatase, Q9VWE4_DROME vs Q4DKS2_TRYCC *(TcCLB.509569.160)*. (B) Ubiquinol-cytochrome c chaperone (CBP3), P21560 CBP3_YEAST vs A4I4I7_LEIIN *(LINF_290018900)*. (C) Transcription initiation factor TFIID subunit 2, A0A1D8PQF6_CANAL vs E9AGI7_LEIIN *(LINF_120016700*). (D) E3 ubiquitin-protein transferase MAEA, Q7L5Y9 MAEA_HUMAN vs Q4D4T7_TRYCC *(TcCLB.504253.20).*

We ran the BUSCO eukaryotic database (Benchmarking Universal Single-Copy Orthologs algorithm) [38] for all available protein sequences in TriTrypDB. This database contains 255 “BUSCO groups”, corresponding to eukaryotic single copy ortholog genes common to most organisms considered to construct the database. The analysis resulted in 68 groups that were reported as “Missing” in TriTrypDB. Indicating that no kinetoplastid protein queries matched the HMM profiles of these groups, meaning that these proteins are either missing, very fragmented or too divergent to produce a significant hit [38]. Our structure comparison approach identified putative homologs for 48 out of these 68 missing BUSCO groups. Notably, 39 of these genes were annotated in TriTrypDB or UniProt, likely through other annotation methods such as experimental evidence, other BUSCO databases or annotation tools. The remaining 9 BUSCO groups were identified by using our SRBH approach; examples are provided in Figs 5 and S3. Gene IDs for all proteins belonging to these clusters are provided in S3 and S4 Tables. These genes span distinct core functional categories, including transcription, DNA repair, translation, and cell cycle regulation. We conducted a detailed analysis of these genes by characterizing their domain architecture using InterProScan, identifying BLASTP hits against the complete GenBank non-redundant database (excluding Discoba), and comparing these findings with our annotation transfer from SRBH.

We identified a Dark Cluster represented by *TcCLB.509569.160* as Ssu72 structural homolog, a highly conserved phosphatase critical for transcriptional regulation (Fig 5A). Ssu72 targets the C-terminal domain (CTD) of RNA polymerase II, which is essential for transcription initiation, elongation, and termination [39]. Interestingly, the current model of the cleavage and polyadenylation complex (CPF) in kinetoplastids (based on a *T. brucei* study [40]) supports that it is largely conventional [41], except for two trypanosome-specific subunits (*Tb927.11.13860* and *Tb927.8.4480*, found by tandem affinity purification of CPF) [40] and no potential CTD phosphatase among the CPF components [41]. Our analysis reveals that *Tb927.8.4480* is a member of *TcCLB.509569.160* cluster (S3 Table) and subsequently Ssu72 structural homolog. Regarding *Tb927.11.13860* it has SRBH to CSRP1 (Cysteine and glycine-rich protein 1). Although they have been found associated with CPF, these two are the only ones that no homology could be assigned through comparative analysis and sequence homology-based searches [40]. Experimental results indicates that *Tb927.8.4480* appears to play a repressive role in polyadenylation, with its depletion moderately affecting spliced-leader RNA accumulation and trans-splicing, suggesting its involvement in interconnected mRNA processing steps [40]. On the other hand, our knowledge of *Tb927.11.13860* (CSRP1) remains limited; however, the identification of homologs suggests that it is unlikely to be a species-specific protein. The characteristic InterPro domain IPR006811, which spans almost the entire length of the protein, is detected in all reference organisms and is the only identified domain. However, this domain is absent in all members of the *TcCLB.509569.160/Tb927.8.4480* cluster (Fig 5B), and no other InterPro domains are detected (S6 Table). Consistently, no BLASTP hits were found in the GenBank non-redundant database.

Also related with transcription we found homologs of General Transcription and DNA Repair Factor IIH subunit TFB4 (TFB4) (S3C Fig), a crucial component of the TFIIH core complex involved in DNA damage response, repair, and transcription regulation [42]. Two kinetoplastid clusters showed SRBH with TFB4. One cluster, represented by *Lsey_0010_0360*, is a Dark Cluster, while the other, represented by *TcCLB.508707.149*, contains multiple members annotated as “TFIIH basal transcription factor subunit” in TriTrypDB. This annotation is based on experimental studies in *T. brucei*, where tandem affinity purification followed by mass spectrometry and supervised sequence analysis identified *Tb927.11.16070* as TFB4 [43]. Notably, the authors reported that BLAST searches failed to detect homology outside trypanosomatids. Although this *T. brucei* gene is absent from our identified clusters, both clusters and *Tb927.11.16070* belong to the same orthologous group defined by OrthoMCL (OG6_158123). The reference organism’s TFB4 is characterized by the ‘TFIIH subunit Tfb4/GTF2H3’ (IPR004600) and ‘von Willebrand factor A-like domain superfamily’ (IPR036465) InterPro domains (S6 Table). However, no InterPro domains were detected in the kinetoplastid sequences of the clusters that were identified by structural similarity (S6 Table), and no BLASTP hits were obtained, highlighting the ability of SRBH to automatically infer biologically relevant relationships. Regarding proteins related to protein assembly and mitochondrial functions, we found SRBH to the Ubiquinol-Cytochrome C Chaperone (CBP3), a protein vital for the assembly of Ubiquinol-cytochrome C Reductase in the mitochondrial respiratory chain [44], and the Iron-Sulfur Cluster Co-Chaperone Protein HscB is involved in iron-sulfur cluster assembly and protein maturation [45,46], contributing to mitochondrial electron transport chain function. For all the members of the cluster that is represented by *LINF_290018900* (Fig 5B), no domains were recognized by InterProScan (S6 Table), including the distinctive domains of CBP3 (“Ubiquinol-cytochrome c chaperone, CBP3” (IPR007129) and “Ubiquinol-cytochrome c chaperone” (IPR021150)) that are present in all their SRBH top hits (S6 Table). No significant BLASTP hits to the GenBank non-redundant database were obtained.

These examples illustrate how our approach can establish remote homology where conventional methods fail. In two cases, experimental evidence was crucial: for TFB4, precise identification was achieved through experimental results and detailed sequence analysis, while for Ssu72, its experimental association with CPF did not reveal any known homologs, leading to its classification as kinetoplastid-specific. In contrast, CBP3 lacks experimental validation, yet our pipeline strongly supports its proposed identity. These findings highlight the power of our method in resolving challenging annotation cases in an unsupervised manner, assigning homology to proteins previously considered hypothetical or species-specific, and uncovering new insights into their evolutionary and functional roles.

For subsequent cases, while standard sequence analyses and expert curation may provide partial or complete annotations, our pipeline serves as a powerful complementary tool for elucidating homology and, ideally, the function of kinetoplastid genes.

Regarding transcription-related proteins, we identified structural homologs of Transcription Initiation Factor TFIID Subunit 2 (TAF2), a key component of the TFIID complex essential for initiating DNA-dependent RNA polymerase II transcription. The representative of the kinetoplastid cluster, *LINF_120016700*, is currently annotated as a “puromycin-sensitive aminopeptidase-like protein” (Fig 5C). Our SRBH approach suggests that *LINF_120016700* (E9AGI7_LEIIN) shares structural similarity with *TAF2* across multiple reference organisms (S5 Table). However, HMM-based domain annotation alone can only confidently place these proteins within the same family or superfamily, as both contain the domains “Peptidase M4/M1, CTD superfamily” (IPR027268) and “Aminopeptidase N-like, N-terminal domain superfamily” (IPR042097). Notably, the *TAF2*-specific InterPro domain (IPR037813) is absent in all sequences of the *LINF_120016700*/E9AGI7_LEIIN cluster (S6 Table). Complementary BLASTP analyses against the NCBI nr database yielded significant e-values (~1e–60) for sequences annotated as “puromycin-sensitive aminopeptidase”. However, these hits are confined to the N-terminal ~500 aa region (~40% coverage), corresponding to the Aminopeptidase N-type domain, with only ~30% sequence identity. This highlights the limitations of sequence-based methods in identifying homologs of highly divergent proteins, which our structural homology approach associated with TAF2.Additionally, and related to transcription, we found two clusters represented by *LINF_320046700* and *TcCLB.504005.50* as homologs of the Cleavage Stimulation Factor subunit 3 (CSTF3) (S3E Fig), a protein required for the polyadenylation and 3′‐end cleavage of mammalian pre‐mRNAs [36,37]. The InterPro database includes an HMM for CSTF3, named “mRNA 3′‐end‐processing protein Rna14‐like” (IPR045243). Both kinetoplastid clusters are recognized by IPR045243; however, the UniProt automatic annotation algorithm does not assign a corresponding gene name. Moreover, as a reference, all top hits in reference organisms contain the “Suppressor of forked” domain (IPR008847), which is not detected in our clusters (S6 Table). In these cases, our approach provides additional evidence for the identity, although it is not as essential for inferring homology as in the above examples. BLASTP results yielded few hits, with low taxonomic diversity, approximately 20% coverage (matching the “Suppressor of forked” domain), ~ 25% sequence identity, and e-values above 1e-6. Most hits lacked annotations, except for one in *Tieghemostelium lacteum* (KYQ88973.1), labeled as “cleavage stimulation factor subunit 3.”

Related to translation, we report structural homologs to the tRNA (guanine-N(7)-)-methyltransferase non-catalytic subunit (Trm82) (S3D Fig) which plays a role in tRNA and mRNA processing [47]. The kinetoplastid sequences associated with this gene are found in Dark Clusters, except for one sequence with partial information. This represents an interesting case, as the specific HMM (IPR028884) that is recognized in Trm82 also is identified in members of these clusters (S6 Table), and BLASTP results corroborate this annotation, even though the statistical parameters fall in the gray zone to consider a significant BLASTP hit to determine homology.

Regarding protein assembly, we identified homologs to Hsc20/HscB/Jac1 co‐chaperone. In this case, three kinetoplastid clusters report SRBH with TM‐scores between 0.6-0.7 (represented by *LtaPh_3329200*, *LmjF.25.1690*, and *Tb927.3.1760*). In TriTrypDB, members of these clusters are annotated at the superfamily level as “J domain‐containing protein” or equivalent. In reference organisms, Hsc20/HscB/Jac1 includes two distinct HMMs: HscB_oligo_C (IPR009073) and HscB (IPR004640). Notably, in *LtaPh_3329200* cluster members these distinctive HMMs were not detected, whereas in the other two clusters the HscB (IPR004640) was successfully recognized (S6 Table). BLASTP results yielded hits labeled as “J domain-containing protein” for *LtaPh_3329200* and “Jac1”, and “Fe–S protein assembly co-chaperone HscB” for the other two clusters, with e-values roughly between 1e-5 and 1e-6, coverage ranging from 20% to 80%, and sequence identities around 30–40%. Although these findings support an Hsc20/HscB/Jac1 identity for *LmjF.25.1690*, and *Tb927.3.1760* and superfamily level classification for *LtaPh_3329200*, the overall hit qualities are moderate.

Two clusters were identified in “Golgi to ER traffic protein 4” (GET4) BUSCO group by SRBH. The first, represented by *TcCLB.510187.140*, yielded no BLASTP hits in NCBI and no domains were detected by InterProScan (S6 Table), despite its description as “hypothetical protein – conserved” in TriTrypDB; its top SRBH results include four out of five proteins annotated as “Golgi to ER traffic protein 4 homolog” (IPR007317) with TM‐scores around 0.7. The second cluster, represented by *LtaPh_3406000*, does not yield BLASTP hits but contains a significant match to the HMM for “NSF attachment protein” (IPR000744) (S6 Table). Consistently, many cluster members are annotated as “Gamma‐soluble NSF attachment protein (SNAP‐gamma)”. In our results, three of the four top hits contain the GET4 domain (IPR007317), while the remaining one is annotated as “Gamma‐soluble NSF attachment protein”. Notably, both the “NSF attachment protein” and GET4 domains belong to the Tetratricopeptide‐like helical domain superfamily (IPR011990). This is the only case among our “case study” where annotation ambiguities were encountered, indicating that further detailed analysis is warranted.

For cell cycle regulation, we found the E3 Ubiquitin-Protein Transferase MAEA (MAEA) (Fig 5D), a core component of the CTLH E3 ubiquitin-protein ligase complex, which is crucial for ubiquitination, proteasomal degradation, and cell proliferation [48]. For the identification of MAEA, important domains such as CTLH_C (IPR006595), CTLH/CRA (IPR024964), CRA_dom (IPR013144), and ZF_RING_GID (IPR044063) should be detected; however, these domains are absent in all members of *TcCLB.504253.20* cluster. Notably, we found by InterProScan analysis an informative and specific HMM for *MAEA* (IPR045098) that covers the entire sequence, supporting the same identity as that proposed by our approach (S6 Table). No BLASTP hits were obtained against NCBI.

Of these 9 BUSCO groups, 4 had SRBHs with a single kinetoplastid cluster within our database (Fig 5), while 5 had multiple SRBHs (S3 Fig). Examining the four sequence clusters with multiple SRBHs, we observe that the clusters are grouped by genus, with Trypanosoma generally being distinct from the other genera (S5 Table). This is primarily due to the stringent criteria regarding sequence identity and coverage used for clustering kinetoplastid proteins sequences, often resulting in the division of homologous genes into subclusters. If only one target reference organism was used, only one of the clusters would represent the SRBH. However, by using several reference organisms, we obtained multiple SRBHs for the same BUSCO group or gene homologs. None of the structural alignments in Figs 5 and S3 exceeded a sequence identity of 20% (except for some hits from co-chaperone Hsc20), demonstrating the high level of sequence divergence and, consequently, the difficulty in establishing homology relationships through standard sequence-based methods (see S5 Table). The subcellular locations, according to TrypTag, of the “case study” proteins are shown in S4 Fig and are consistent with our results.

## Discussion

The field of genomic homology detection and the possibility to transfer functional annotation from structural homologs, is undergoing a paradigm shift, partly due to the emergence of methods that reveal remote homology between genes [10]. Specifically, the advent of algorithms for predicting tertiary structure and conducting efficient structural comparisons has introduced a novel approach to assessing protein similarity [12–15]. It has been demonstrated that sequences-dependent bioinformatics methods cannot annotate essential eukaryotic proteins in cases of high degree of sequence divergence [10,49,50]. Therefore, developing new methods based on structural comparison known to overcome these limitations holds great potential for assigning homology and putative functions to many currently unannotated genes through homology inference to highly divergent genes [10,17,51,52]. While the strengths and limitations of these methods are currently being evaluated, they have proven to be valuable tools for elucidating homology and gene family classification of previously uncharacterized proteins [10,17,52]. Kinetoplastids offer a unique opportunity for these approaches due to their early divergence in the eukaryotic tree, the significant divergence between groups (e.g., Leishmania and Trypanosoma), and the limited scope of current annotation.

The bioinformatic pipeline described here (ASC) aims to facilitate structural inference of homology, allowing annotation transfer from sequence divergent homologous proteins. Even though this method might produce false positive functional assignments caused by functional divergence of structural homologs, this strategy has been shown to be accurate in recent publications [10,17,18,52]. ASC relies on SRBH instead of all best hits creating a stringent criterion for identifying homologous proteins and increasing precision [17]. As this results in lower sensitivity there is still room to delve deeper into the annotation by compromising some of the specificity given by the SRBH strategy. The strategy requires a database of protein structures from reference organisms making the selection crucial; in this work we used species representative of diverse evolutionary lineages.

To increase computational efficiency, we select a representative homolog from highly similar protein for the subsequent structural homology search. The high level of divergence within kinetoplastids explains that during the initial clustering step, we noticed the sub-clustering of homologous genes within kinetoplastids. The comparison of each representative to each reference organism separately compensates for some of the issues raised by this in the context of RBH strategy. Lowering the sequence identity threshold to cluster more dissimilar sequences could partially solve this issue but this would come with an increase in false positive homology detection (20–35% sequence identity) [53] and was not deemed appropriate. Although adding a structure-level clustering step after sequence-level clustering could also address this, it would be computationally expensive, even though this can be implemented in future pipeline versions.

An intrinsic limitation of our approach relies on the fact that kinetoplastids are not part of the reference organisms set, so our approach will not yield results for kinetoplastid-specific proteins, such as those coding for surface protein families. A perspective related to performing structure-based comparisons within kinetoplastids, is the identification of homologs and, eventually, orthologs, enhancing future phylogenetic studies and comparative genomics among this group. This is particularly true for identifying genes with greater divergence or rate of evolution and the processes underlying them.

In the current work, we applied the ASC pipeline to all available kinetoplastid genomes. Considering proteins named as hypothetical in the reference TriTrypDB database, that passed the sequence-based clustering and cluster selection steps, our work was able to find homologs to most of them within kinetoplastids. Among these, many were part of clusters where at least one member already had prior annotations. Therefore, for this group, our pipeline can be used to increase the functional annotation of current uncharacterized proteins by transferring information from homologous genes determined by sequence similarity within kinetoplastid genomes. The remaining comprised uncharacterized proteins that were found in Dark Clusters that matched known structures in reference organisms. This group totals around 23,000 kinetoplastid proteins from 942 clusters. After retrieving all available database information, reanalyzing these proteins using sequence-based methods, and transferring annotations within clusters, 6,700 proteins from 310 clusters remained without any biological information. We consider this number to be significant, underscoring the utility of the structural homology search approach for kinetoplastid protein-coding genes. Even when the functional annotation of the reference organisms protein hit is limited, a match suggests that the kinetoplastid hypothetical protein is not an annotation error and points to the protein’s relevance to the parasite, given its structural conservation.

It is worth noting that validating *in silico* genome annotation of dark matter proteins presents significant challenges due to the absence of a broad and definitive gold standard for comparison [54]. Our strategy employed two alternative approaches. First, we analyzed the currently annotated kinetoplastid proteins. This shows that the transfer of annotation from the SRBH obtained by our approach aligns well with current annotation of kinetoplastid genomes. Furthermore, our results reveal that the transfer of putative annotation from structural homologs often provides a deeper functional understanding by assigning new InterPro and Pfam domains to currently annotated genes. This additional information enhances our knowledge beyond what databases currently provide. It is important to note that these comparisons rely on the current annotation of kinetoplastid proteins in the database, as re-annotating the entire database using sequence-based methods is beyond the scope of this manuscript. Second, we leveraged TrypTag, a genome-wide experimental database of protein subcellular locations, achieving similar results. This demonstrates that our approach yielded comparable or superior results to existing automated annotation methods. Remarkably, our method was capable of transferring homology from reference organisms to a significant number of currently unannotated proteins present in Dark Clusters of kinetoplastid genomes.

Building on our results, we investigated whether we could use our results to shed light into conserved, and thus, putative functionally relevant eukaryotic proteins that are challenging to identify in kinetoplastid genomes by sequence-based homology inference methods. Our hypothesis was that some of these functions are carried out by genes with high sequence divergence. To address this, we employed BUSCO to identify single copy conserved eukaryotic genes not detected by this tool in kinetoplastid genomes. The combination of the tool with our results allowed us to identify homologs for nine groups with ubiquitous representation in eukaryotic genomes, that are challenging to identify otherwise. These groups encompass a range of relevant molecular processes, including transcription, translation, DNA repair, and cell cycle regulation. Our results further support the significance of employing structural comparisons in divergent organisms.

## Materials and methods

### Kinetoplastid protein sequences and structures

We manually downloaded all the predicted protein sequences and annotations available in TriTrypDB release 65 (S1 Table) [27]. In this work, proteins were classified as hypothetical based on the gene description field provided by the database. TriTrypDB currently relies on the EBI pipeline for genome sequence analysis and annotation. This pipeline performs sequence-based homology searches using InterProScan against InterPro member databases, retrieving information as InterPro entry descriptions, domain structure and GO terms.

We clustered the sequences using MMseq2 with “foldseek cluster” (sensitive clustering) and parameters “--cluster-mode 1 --similarity-type 2 --min-seq-id 0.5 -c 0.8 --cov-mode 0 -e 1e-5” [18,29]. We considered all protein sequences in each cluster as homologs. Clusters with less than ten protein sequences were discarded. All available structures for each sequence in the remaining clusters were obtained via FTP from the AFDB. Since clusters can contain multiple structures, we calculated the average pLDDT for each structure and selected the one with the highest as the cluster representative [29] (using in-house Python scripts available on https://github.com/JuanTrinidad/ASC).

As inconsistencies between the sequences from TriTrypDB and AFDB were observed for some proteins, structure files were curated by comparing their sequence to the one from TriTrypDB. If the length of the protein in the PDB file and the TriTrypDB protein sequences were identical, they were deemed matching. Conversely, both protein sequences were aligned if a length discrepancy was observed. Sequences exhibiting less than 80% identity were excluded from consideration.

### Proteins structure data from reference organisms

The protein structures of reference organisms used in this work were downloaded from “AFDB model organism proteomes” (https://ftp.ebi.ac.uk/pub/databases/alphafold/v4/). Kinetoplastid proteomes were excluded, obtaining a final number of 45 proteomes (S2 Table).

### Protein structure comparison

Protein structures were compared using Foldseek release 8.ef4e960 [12]. First, query (14,267 protein structures representative of all kinetoplastid clusters) and target (one for each independent reference organism) databases were created using the “foldseek createdb” command. RBH comparisons were performed independently between the query database and each target database using “foldseek rbh” with parameters “-s 9.5 -c 0 -a”.

Structural alignment was performed using the FATCAT algorithm with default parameters (Flexible structure AlignmenT by Chaining Aligned fragment pairs, allowing Twists) [23,25]. The TM-scores for the comparison of “Chain 1” versus “Chain 2” (and vice versa) were extracted from the FATCAT results. Alignments were visualized using ChimeraX software [55].

### Validation of inferred annotation

To validate our results, for all kinetoplastid cluster representatives and all reference organism proteins, we downloaded from UniProt the protein annotation of the Protein Families and InterPro including Pfam and PANTHER, among other databases [26]. For each SRBH, when available, we compared query and target annotation by creating five categories. Sets are defined as K (set of annotations for the query kinetoplastid SRBH protein) and RO (set of annotations for the reference organism protein).

Categories:

- ALL: All terms match (K = RO)
- K_in_RO: some terms of the kinetoplastid query are present in the list of terms of the model organism protein (K ⊆ RO)
- RO_in_K: some terms of the model organism protein are present in the list of terms of the kinetoplastid query protein (RO ⊆ K)
- PARTIAL: terms of the kinetoplastid query and terms of the model organism protein partially intersect ((K ∩ RO) ≠ Ø and K ≠ RO and RO ≠ K)
- ZERO: no matching terms (K ∩ RO = Ø)

Chi-square test for independence was performed using the chi2_contingency function from the SciPy library [56] and barplots visualized with the Seaborn library.

Gene Ontology enrichment analysis was performed and visualized by WEGO 2.0 (Web Gene Ontology Annotation Plot) [57].

### Validation of protein subcellular localization prediction using TrypTag

Our results were also evaluated using the TrypTag database [36], which has experimental protein targeting and localization data for almost all protein coding genes in *T. brucei*.

We downloaded the subcellular localization data (Gene Ontology, cellular component) from UniProt [26] and the experimental data of TrypTag from TriTrypDB. We selected nine ubiquitous subcellular localizations for the comparison: cytoplasm, nucleoplasm, nucleolus, basal body, endoplasmic reticulum, mitochondrion, nuclear envelope, nucleus, and Golgi apparatus. We compared the semantic similarity between GO terms from UniProt and TrypTag using GOGO [37]. Before the comparison, GO terms from TrypTag with percentages or weak annotations “weak|10%|25%|50%|75%” were considered low-confidence annotations and removed. Given that many genes possess multiple ontology terms within the UniProt annotation (frequently nested), we conducted a comprehensive comparison against the TrypTag annotation. For each gene, using GOGO, each Uniprot GO term obtained by structural annotation was compared to all GO terms annotated in TrypTag. We defined the GOGO score between both annotations as the highest score obtained in each comparison.

The comparisons of score distributions from TrypTag validation were tested using the Mann-Whitney-U tests from the SciPy library [56] and visualized with the Seaborn library.

### BUSCO analysis

We used the BUSCO [38] to identify conserved eukaryotic genes that this tool reports as missing in kinetoplastids. For this, we ran BUSCO on the TriTrypDB protein fasta file using the “-m prot” option and the eukaryota_odb10 database. For comparison, we performed the same procedure for model organism proteomes.

### Protein domains identification

We used InterProScan version 5.67-99.0 [20] with default parameters to analyze protein sequences within “Dark Clusters”. For comparison, the same procedure was applied to the protein sequences of the top hits from the SRBH reference organisms.

### BLASTP homology searches

BLASTP searches were performed using the NCBI BLASTP server with default parameters. The representative sequences of each cluster were used as queries against the non-redundant protein sequences (nr) database. Additionally, taxonomic filtering was applied to exclude sequences from Discoba (taxid:2611352).

## Supporting information

S1 Fig Gene ontology terms enrichment analysis in the 942 dark clusters. Gene ontology term enrichment analysis performed with WEGO2.0 comparing the 942 Dark Clusters annotated by our method against all clusters. The analysis includes biological processes, molecular functions, and cellular components categories.
(TIF)

S2 Fig Comparison of GO enrichment analysis between GO terms from the current annotation and ASC-derived GO terms. *Upper Panel:* RiboSeq analysis for *T. brucei* [35]. Up-regulated genes from the comparison between in-vivo derived slender bloodstream forms and cultured procyclic (insect midgut) forms were analyzed. *Lower Panel:* RiboSeq analysis for *T. cruzi* [34]. Up-regulated genes from the comparison between non-infective (epimastigote) and infective (metacyclic trypomastigote) forms were analyzed.
(TIF)

S3 Fig Conserved eukaryotic proteins annotated by our approach that have been divided into subclusters by MMseqs2. The figure shows “BUSCO groups” that obtained more than one SRBH with the kinetoplastid protein clusters. The target protein (model species) is shown in violet, and the query protein (kinetoplastids) is shown in pink. In most cases, when SRBH identified homologs of the protein in multiple organisms, we selected one as a representative example. Target structures shown in the comparisons are highlighted in bold (A) Golgi to ER traffic protein 4. a1. Kinetoplastid structure: LtaPh_3406000 (A0A640KRB8), Target structures: AJECG_C0P182, PARBA_C1GXR3, SPOS1_U7PSW4. a2. Kinetoplastid structure: TcCLB.510187.140 (A0A2V2WCY5), Target structures: BRUMA_A0A0K0K0X7, CANAL_A0A1D8PND7, GET4_DICDI, PLAF7_Q8IL82. (B) Co-chaperone Hsc20. b1. Kinetoplastids structures: Tb927.3.1760 (Q57ZD5). Target structures: ORYSJ_Q2QRM4, b2. Kinetoplastids structures: LmjF.25.1690 (Q4Q9S1), Target structures: DROME_A8JNT7, RAT_D3ZME7, HUMAN_Q8IWL3, ARATH_Q8L7K4, b3. Kinetoplastids structures: LtaPh_3329200 (A0A640KWU3), Target structures: AJECG_C0NFZ6, PARBA_C1H2E3. (C) TFIIH subunit Tfb4/GTF2H3. c1. Kinetoplastids structures: TcCLB.508707.149 (Q4E262), Target structures: SOYBN_A0A0R0JLP0, SCHPO_O74366, YEAST_Q12004, c2. Kinetoplastids structures: Lsey_0010_0360 (A0A0N1IME3), Target structures: AJECG_C0NNU2. (D) tRNA (guanine-N(7)-)-methyltransferase non-catalytic subunit. d1. Kinetoplastids structures: TcCLB.507711.120 (Q4DZR6), Target structures: DANRE_A4IGH4, SCHPO_O74863, YEAST_Q03774, MOUSE_Q9EP82, d2. Kinetoplastids structures: TcIL3000_10_10140 (G0UXW8), Target structures: CANAL_Q5AH60. (E) Cleavage stimulation factor subunit 3. e1. Kinetoplastids structures: TcCLB.504005.50 (Q4CY43), Target structures: SCHMA_A0A3Q0KPI3, RAT_F1M4W7, e2. Kinetoplastids structures: LINF_320046700 (A4CY43), Target structures: DANRE_F1QIB2, HUMAN_Q12996, MOUSE_Q99LI7.
(TIF)

S4 Fig Subcellular Localization of selected *T. brucei* proteins in TrypTag. Fluorescence microscopy images of *T. brucei* proteins (GFP tagged) from the case study clusters, showing their subcellular localization as determined by TrypTag. *Upper panel*: *Left*: Tb927.4.2890: Cytoplasm (weak; points), E3 ubiquitin-protein transferase MAEA (Q4D4T7) *Right*: Tb927.8.4480: Nucleoplasm, RNA polymerase II subunit A C-terminal domain phosphatase SSU72 (Q4DKS2). *Lower panel*: Tb927.3.1760: Cytoplasm (points), Co-chaperone Hsc20 family protein (Q57ZD5).
(TIF)

S1 Table Genome descriptions of kinetoplastids used in this work. (PDF)

S2 Table Proteome descriptions of reference organisms used in this work. (PDF)

S3 Table Gene IDs of kinetoplastid genes in the 4 BUSCO conserved protein clusters with a single SRBH. (PDF)

S4 Table Gene IDs of kinetoplastid genes that belong to clusters sharing the same hit in the SRBH search. (PDF)

S5 Table Detailed information of “case study” SRBH results. (PDF)

S6 Table Protein domains identified by InterProScan. (PDF)
