# Supplementary figures and images for: Expanding kinetoplastid genome annotation through protein structure comparison
Source: PLoS Pathog. 2025 Apr 21;21(4):e1013120. doi: 10.1371/journal.ppat.1013120 (PMC12047770; doi:10.1371/journal.ppat.1013120)

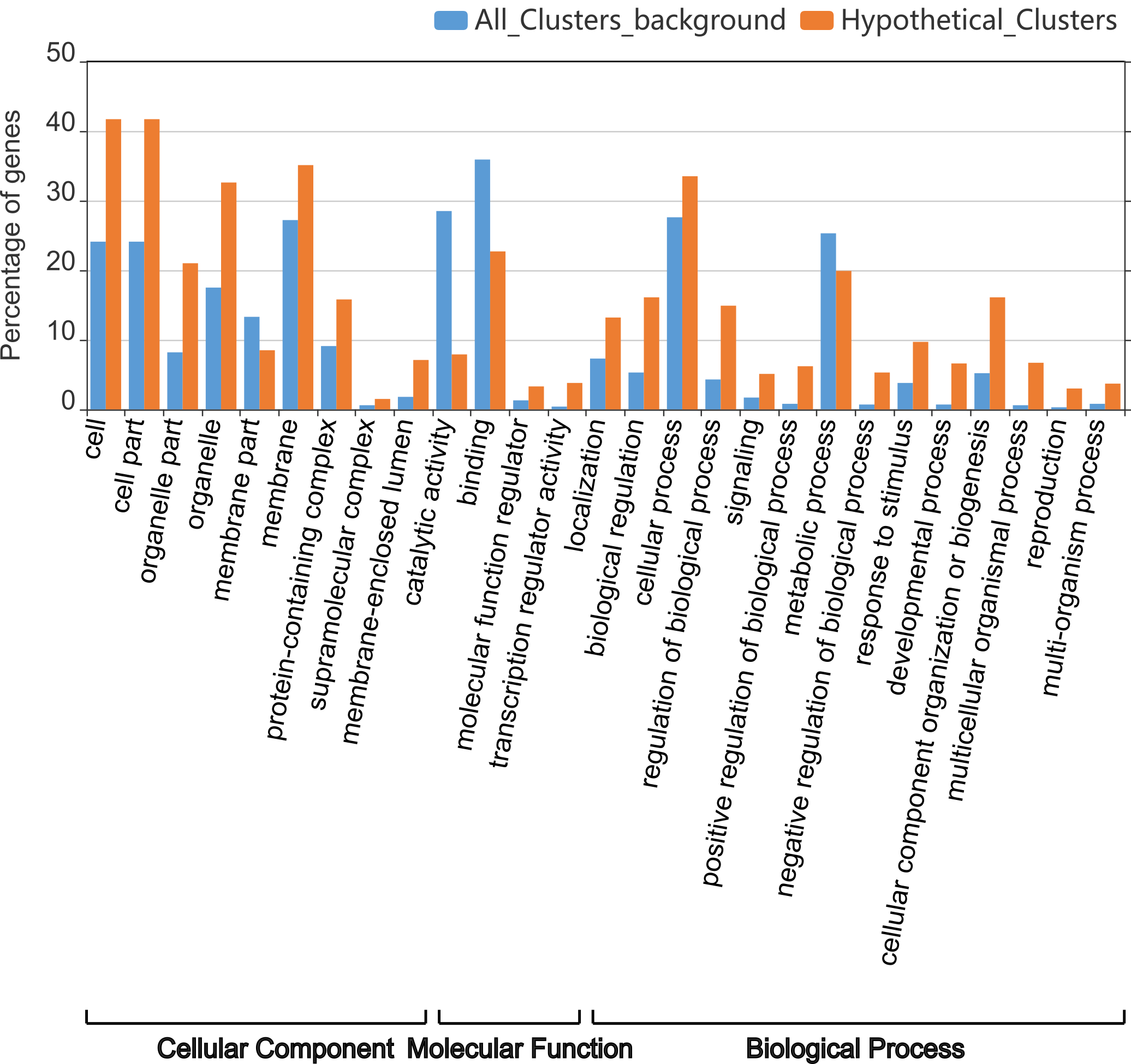

Supplement: S1 Fig — Gene ontology term enrichment analysis performed with WEGO2.0 comparing the 942 Dark Clusters annotated by our method against all clusters. The analysis includes biological processes, molecular functions, and cellular components categories. (TIF) [file ppat.1013120.s001.tif]

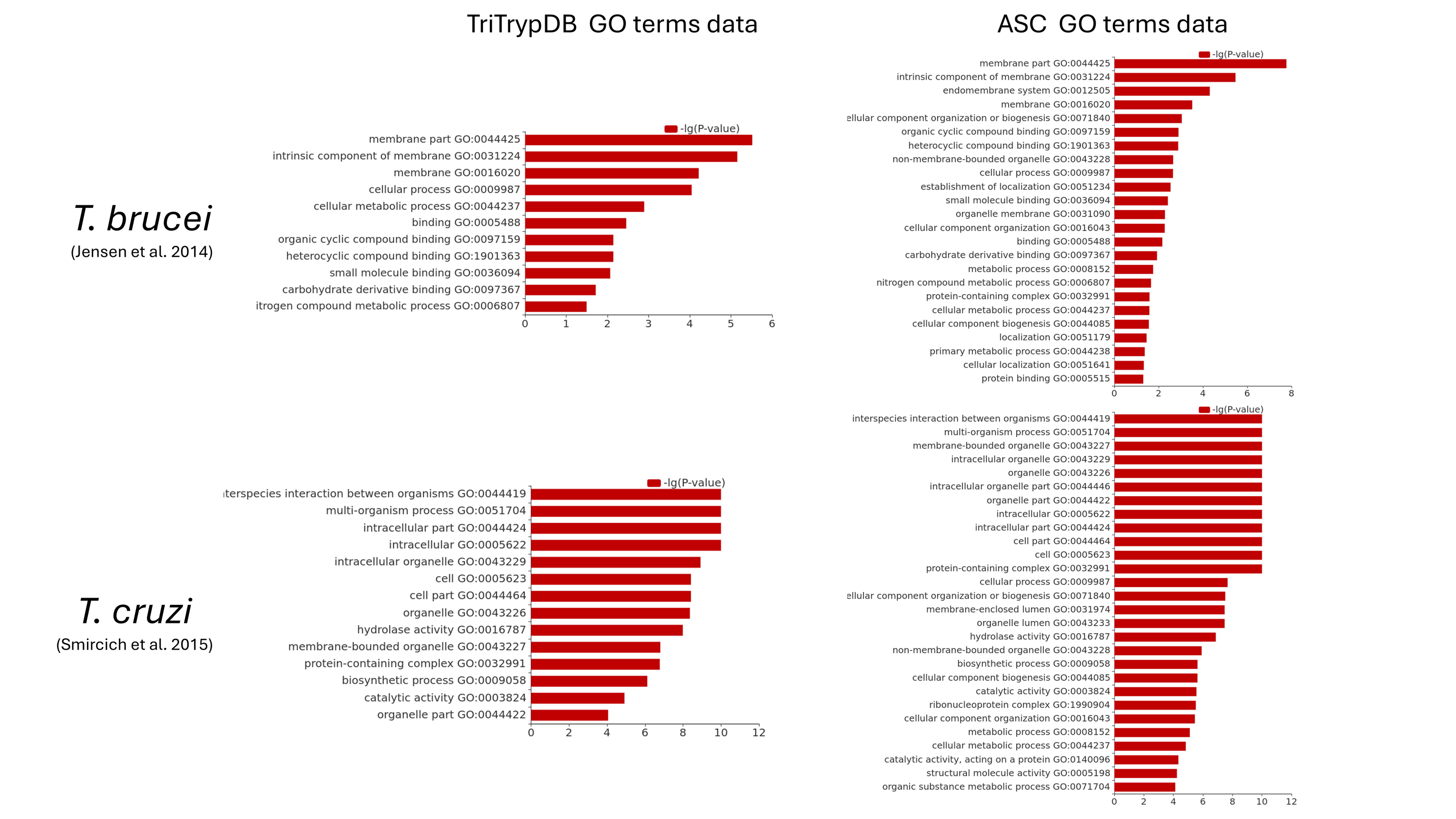

Supplement: S2 Fig — Upper Panel: RiboSeq analysis for T. brucei [35]. Up-regulated genes from the comparison between in-vivo derived slender bloodstream forms and cultured procyclic (insect midgut) forms were analyzed. Lower Panel: RiboSeq analysis for T. cruzi [34]. Up-regulated genes from the comparison between non-infective (epimastigote) and infective (metacyclic trypomastigote) forms were analyzed. (TIF) [file ppat.1013120.s002.tif]

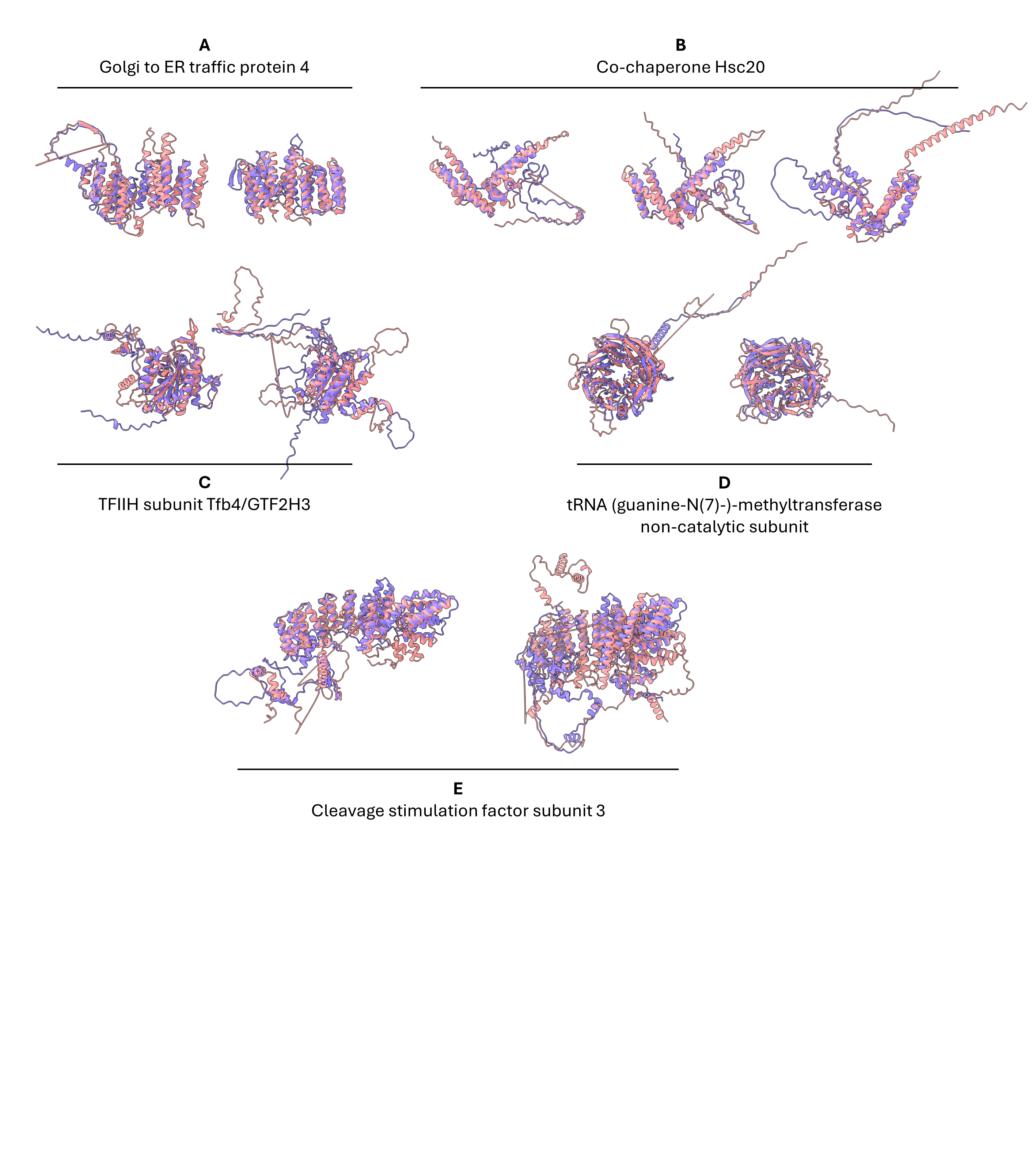

Supplement: S3 Fig — The figure shows “BUSCO groups” that obtained more than one SRBH with the kinetoplastid protein clusters. The target protein (model species) is shown in violet, and the query protein (kinetoplastids) is shown in pink. In most cases, when SRBH identified homologs of the protein in multiple organisms, we selected one as a representative example. Target structures shown in the comparisons are highlighted in bold (A) Golgi to ER traffic protein 4. a1. Kinetoplastid structure: LtaPh_3406000 (A0A640KRB8), Target structures: AJECG_C0P182, PARBA_C1GXR3, SPOS1_U7PSW4. a2. Kinetoplastid structure: TcCLB.510187.140 (A0A2V2WCY5), Target structures: BRUMA_A0A0K0K0X7, CANAL_A0A1D8PND7, GET4_DICDI, PLAF7_Q8IL82. (B) Co-chaperone Hsc20. b1. Kinetoplastids structures: Tb927.3.1760 (Q57ZD5). Target structures: ORYSJ_Q2QRM4, b2. Kinetoplastids structures: LmjF.25.1690 (Q4Q9S1), Target structures: DROME_A8JNT7, RAT_D3ZME7, HUMAN_Q8IWL3, ARATH_Q8L7K4, b3. Kinetoplastids structures: LtaPh_3329200 (A0A640KWU3), Target structures: AJECG_C0NFZ6, PARBA_C1H2E3. (C) TFIIH subunit Tfb4/GTF2H3. c1. Kinetoplastids structures: TcCLB.508707.149 (Q4E262), Target structures: SOYBN_A0A0R0JLP0, SCHPO_O74366, YEAST_Q12004, c2. Kinetoplastids structures: Lsey_0010_0360 (A0A0N1IME3), Target structures: AJECG_C0NNU2. (D) tRNA (guanine-N(7)-)-methyltransferase non-catalytic subunit. d1. Kinetoplastids structures: TcCLB.507711.120 (Q4DZR6), Target structures: DANRE_A4IGH4, SCHPO_O74863, YEAST_Q03774, MOUSE_Q9EP82, d2. Kinetoplastids structures: TcIL3000_10_10140 (G0UXW8), Target structures: CANAL_Q5AH60. (E) Cleavage stimulation factor subunit 3. e1. Kinetoplastids structures: TcCLB.504005.50 (Q4CY43), Target structures: SCHMA_A0A3Q0KPI3, RAT_F1M4W7, e2. Kinetoplastids structures: LINF_320046700 (A4CY43), Target structures: DANRE_F1QIB2, HUMAN_Q12996, MOUSE_Q99LI7. (TIF) [file ppat.1013120.s003.tif]

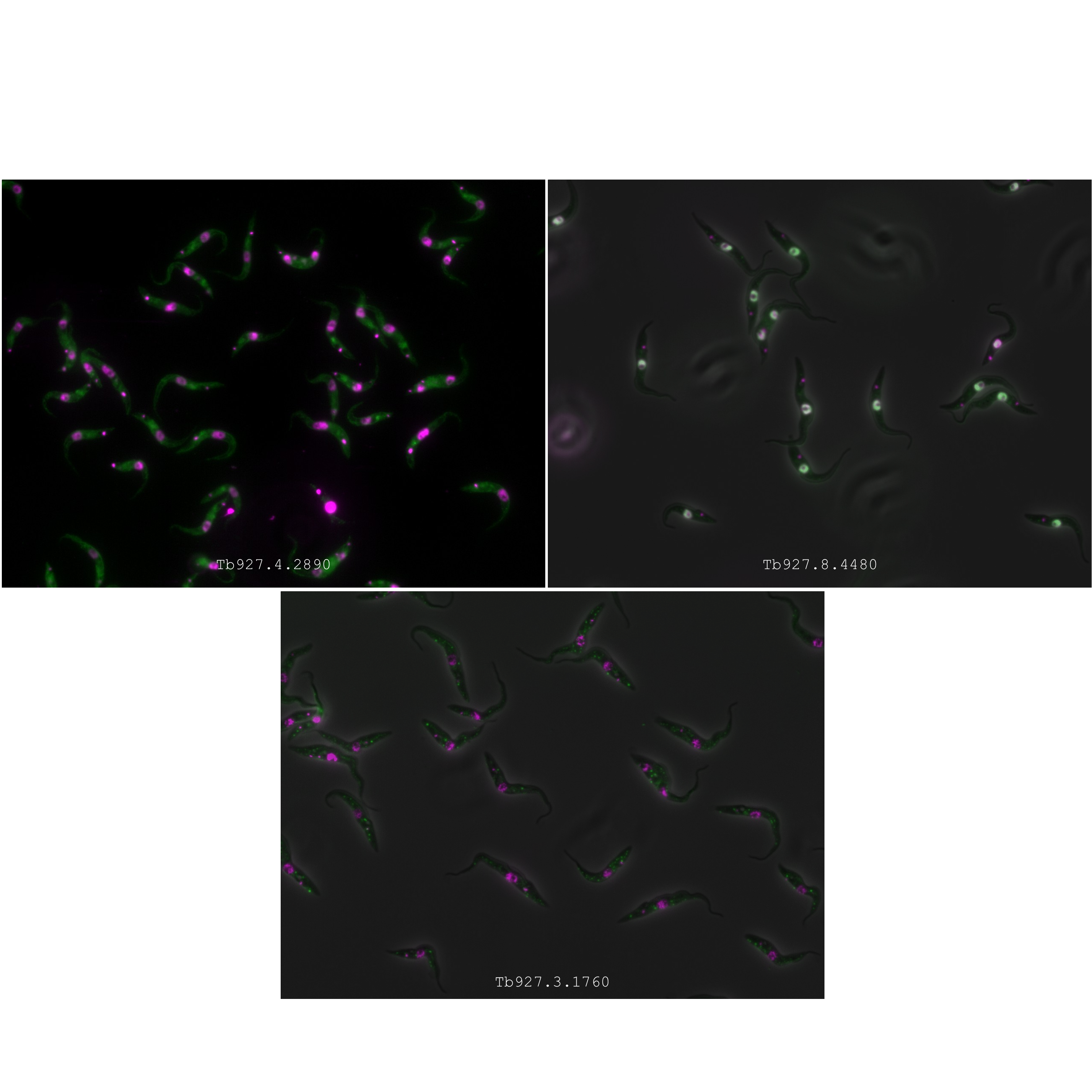

Supplement: S4 Fig — Fluorescence microscopy images of T. brucei proteins (GFP tagged) from the case study clusters, showing their subcellular localization as determined by TrypTag. Upper panel: Left: Tb927.4.2890: Cytoplasm (weak; points), E3 ubiquitin-protein transferase MAEA (Q4D4T7) Right: Tb927.8.4480: Nucleoplasm, RNA polymerase II subunit A C-terminal domain phosphatase SSU72 (Q4DKS2). Lower panel: Tb927.3.1760: Cytoplasm (points), Co-chaperone Hsc20 family protein (Q57ZD5). (TIF) [file ppat.1013120.s004.tif]
