# Supplementary material for: Expanding kinetoplastid genome annotation through protein structure comparison
Source: PLoS Pathog. 2025 Apr 21;21(4):e1013120. doi: 10.1371/journal.ppat.1013120 (PMC12047770; doi:10.1371/journal.ppat.1013120)
Supplement: S1 Table — (PDF) [file ppat.1013120.s005.pdf]

S1 Table. Genomes description of kinetoplastids used in this work.

Organism	Genome Source	Genome Version/Assembly ID	Structural Annotation Version	BUSCO Protein	BUSCO Genomic
Trypanosoma vivax Y486	*GenBank*	*GCA_000227375.1*	*6/6/2016*	*C: 94.6%[S: 94.6%,D: 0.0%],F: 2.3%,M: 3.1%,n:130*	*C: 94.6%[S: 94.6%,D: 0.0%],F: 4.6%,M: 0.8%,n:130*
Trypanosoma congolense IL3000	*GenBank*	*GCA_000227395.2*	*6/6/2016*	*C: 93.8%[S: 89.2%,D: 4.6%],F: 5.4%,M: 0.8%,n:130*	*C: 92.3%[S: 68.5%,D: 23.8%],F: 6.9%,M: 0.8%,n:130*
Leishmania aethiopica L147	*GenBank*	*GCA_000444285.2*	*16/12/2014*	*C: 95.4%[S: 95.4%,D: 0.0%],F: 1.5%,M: 3.1%,n:130*	*C: 100.0%[S: 100.0%,D: 0.0%],F: 0.0%,M: 0.0%,n:130*
Leishmania tropica L590	*GenBank*	*GCA_000410715.1*	*16/12/2014*	*C: 100.0%[S: 98.5%,D: 1.5%],F: 0.0%,M: 0.0%,n:130*	*C: 100.0%[S: 99.2%,D: 0.8%],F: 0.0%,M: 0.0%,n:130*
Leishmania panamensis MHOM/COL/81/L13	*GenBank*	*GCA_000340495.1*	*16/12/2014*	*C: 98.5%[S: 98.5%,D: 0.0%],F: 1.5%,M: 0.0%,n:130*	*C: 100.0%[S: 100.0%,D: 0.0%],F: 0.0%,M: 0.0%,n:130*
Leishmania braziliensis MHOM/BR/75/M2903	*GenBank*	*GCA_000340355.2*	*16/12/2014*	*C: 96.2%[S: 96.2%,D: 0.0%],F: 3.8%,M: 0.0%,n:130*	*C: 97.7%[S: 97.7%,D: 0.0%],F: 2.3%,M: 0.0%,n:130*
Trypanosoma cruzi Dm28c 2014	*GenBank*	*GCA_000496795.1*	*13/11/2013*	*C: 91.5%[S: 91.5%,D: 0.0%],F: 7.7%,M: 0.8%,n:130*	*C: 93.1%[S: 93.1%,D: 0.0%],F: 6.9%,M: 0.0%,n:130*
Trypanosoma brucei brucei TREU927	*GenBank*	*GCA_000002445.1*	*1/11/2020*	*C: 99.2%[S: 93.1%,D: 6.2%],F: 0.0%,M: 0.8%,n:130*	*C: 99.2%[S: 90.0%,D: 9.2%],F: 0.8%,M: 0.0%,n:130*
Leishmania major strain Friedlin	*GenBank*	*GCA_000002725.2*	*28/5/2016*	*C: 100.0%[S: 100.0%,D: 0.0%],F: 0.0%,M: 0.0%,n:130*	*C: 100.0%[S: 100.0%,D: 0.0%],F: 0.0%,M: 0.0%,n:130*
Trypanosoma cruzi strain CL Brener	*GenBank*	*GCA_000209065.1*	*7/12/2015*	*C: 0.0%[S: 0.0%,D: 0.0%],F: 2.3%,M: 97.7%,n:130*	*C: 14.6%[S: 12.3%,D: 2.3%],F: 12.3%,M: 73.1%,n:130*
Leishmania braziliensis MHOM/BR/75/M2904	*GenBank*	*GCA_000002845.2*	*28/5/2016*	*C: 98.5%[S: 98.5%,D: 0.0%],F: 0.8%,M: 0.8%,n:130*	*C: 97.7%[S: 97.7%,D: 0.0%],F: 1.5%,M: 0.8%,n:130*
Trypanosoma rangeli SC58	*GenBank*	*GCA_000492115.1*	*30/10/2013*	*C: 94.6%[S: 94.6%,D: 0.0%],F: 3.8%,M: 1.5%,n:130*	*C: 96.2%[S: 96.2%,D: 0.0%],F: 3.1%,M: 0.8%,n:130*
Leishmania infantum JPCM5	*GenBank*	*GCA_900500625.2*	*13/7/2018*	*C: 100.0%[S: 100.0%,D: 0.0%],F: 0.0%,M: 0.0%,n:130*	*C: 100.0%[S: 100.0%,D: 0.0%],F: 0.0%,M: 0.0%,n:130*
Trypanosoma brucei gambiense DAL972	*GenBank*	*GCA_000210295.1*	*28/5/2016*	*C: 98.5%[S: 98.5%,D: 0.0%],F: 1.5%,M: 0.0%,n:130*	*C: 97.7%[S: 97.7%,D: 0.0%],F: 2.3%,M: 0.0%,n:130*
Leishmania major strain LV39c5	*GenBank*	*GCA_000331345.1*	*16/1/2015*	*C: 100.0%[S: 100.0%,D: 0.0%],F: 0.0%,M: 0.0%,n:130*	*C: 100.0%[S: 100.0%,D: 0.0%],F: 0.0%,M: 0.0%,n:130*
Leishmania major strain SD 75.1	*GenBank*	*GCA_000250755.2*	*16/1/2015*	*C: 98.5%[S: 98.5%,D: 0.0%],F: 0.0%,M: 1.5%,n:130*	*C: 98.5%[S: 98.5%,D: 0.0%],F: 0.0%,M: 1.5%,n:130*
Leishmania mexicana MHOM/GT/2001/U1103	*GenBank*	*GCA_000234665.4*	*28/5/2016*	*C: 99.2%[S: 99.2%,D: 0.0%],F: 0.8%,M: 0.0%,n:130*	*C: 100.0%[S: 100.0%,D: 0.0%],F: 0.0%,M: 0.0%,n:130*
Leishmania donovani BPK282A1	*GenBank*	*GCA_000227135.2*	*28/5/2016*	*C: 99.2%[S: 99.2%,D: 0.0%],F: 0.0%,M: 0.8%,n:130*	*C: 100.0%[S: 100.0%,D: 0.0%],F: 0.0%,M: 0.0%,n:130*
Angomonas deanei strain Cavalho ATCC PRA-265	*INSDC*	*GCA_903995115.1*	*18/8/2020*	*C: 77.7%[S: 77.7%,D: 0.0%],F: 17.7%,M: 4.6%,n:130*	*C: 94.6%[S: 94.6%,D: 0.0%],F: 3.8%,M: 1.5%,n:130*
Blechomonas ayalai B08-376	*GenBank*	*GCA_020509355.1*	*18/1/2017*	*C: 99.2%[S: 99.2%,D: 0.0%],F: 0.8%,M: 0.0%,n:130*	*C: 100.0%[S: 100.0%,D: 0.0%],F: 0.0%,M: 0.0%,n:130*

Bodo saltans strain Lake Konstanz	*GenBank*	*GCA_001460835.1*	*19/11/2015*	*C: 86.9%[S: 84.6%,D: 2.3%],F: 9.2%,M: 3.8%,n:130*	*C: 84.6%[S: 82.3%,D: 2.3%],F: 10.8%,M: 4.6%,n:130*
Crithidia fasciculata strain Cf-Cl	*GenBank*	*GCA_000331325.2*	*1/6/2015*	*C: 96.9%[S: 96.9%,D: 0.0%],F: 1.5%,M: 1.5%,n:130*	*C: 98.5%[S: 86.9%,D: 11.5%],F: 1.5%,M: 0.0%,n:130*
Endotrypanum monterogeii strain LV88	*GenBank*	*GCA_000333855.2*	*16/1/2015*	*C: 97.7%[S: 97.7%,D: 0.0%],F: 2.3%,M: 0.0%,n:130*	*C: 100.0%[S: 100.0%,D: 0.0%],F: 0.0%,M: 0.0%,n:130*
Leishmania amazonensis MHOM/BR/71973/M2269	*GenBank*	*GCA_000438535.1*	*6/12/2018*	*C: 97.7%[S: 97.7%,D: 0.0%],F: 1.5%,M: 0.8%,n:130*	*C: 100.0%[S: 100.0%,D: 0.0%],F: 0.0%,M: 0.0%,n:130*
Leishmania arabica strain LEM1108	*GenBank*	*GCA_000410695.2*	*16/12/2014*	*C: 98.5%[S: 98.5%,D: 0.0%],F: 0.8%,M: 0.8%,n:130*	*C: 100.0%[S: 100.0%,D: 0.0%],F: 0.0%,M: 0.0%,n:130*
Leishmania braziliensis MHOM/BR/75/M2904 2019	*Sanger*	*Jul 30, 2019*	*30/7/2019*		
Leishmania donovani CL-SL	*GenBank*	*GCA_003719575.1*	*9/11/2018*	*C: 100.0%[S: 100.0%,D: 0.0%],F: 0.0%,M: 0.0%,n:130*	*C: 100.0%[S: 100.0%,D: 0.0%],F: 0.0%,M: 0.0%,n:130*
Leishmania donovani HU3	*INSDC*	*GCA_900635355.2*		*C: 99.2%[S: 98.5%,D: 0.8%],F: 0.8%,M: 0.0%,n:130*	*C: 100.0%[S: 100.0%,D: 0.0%],F: 0.0%,M: 0.0%,n:130*
Leishmania donovani strain LV9	*Sanger*	*Feb 27, 2019*	*27/2/2019*		
Leishmania enriettii strain LEM3045	*GenBank*	*GCA_000410755.2*	*16/12/2014*	*C: 98.5%[S: 98.5%,D: 0.0%],F: 0.8%,M: 0.8%,n:130*	*C: 99.2%[S: 99.2%,D: 0.0%],F: 0.8%,M: 0.0%,n:130*
Leishmania enriettii MCAV/BR/2001/CUR178	*INSDC*	*GCA_017916305.1*	*15/4/2021*	*C: 98.5%[S: 98.5%,D: 0.0%],F: 0.0%,M: 1.5%,n:130*	*C: 99.2%[S: 99.2%,D: 0.0%],F: 0.0%,M: 0.8%,n:130*
Leishmania gerbilli strain LEM452	*GenBank*	*GCA_000443025.1*	*16/12/2014*	*C: 100.0%[S: 100.0%,D: 0.0%],F: 0.0%,M: 0.0%,n:130*	*C: 100.0%[S: 100.0%,D: 0.0%],F: 0.0%,M: 0.0%,n:130*
Leishmania major Friedlin 2021	*INSDC*	*GCA_916722125.1*	*3/10/2021*	*C: 100.0%[S: 100.0%,D: 0.0%],F: 0.0%,M: 0.0%,n:130*	*C: 100.0%[S: 100.0%,D: 0.0%],F: 0.0%,M: 0.0%,n:130*
Leishmania martiniquensis LEM2494	*GenBank*	*GCA_000409445.2*	*16/12/2014*	*C: 100.0%[S: 99.2%,D: 0.8%],F: 0.0%,M: 0.0%,n:130*	*C: 100.0%[S: 99.2%,D: 0.8%],F: 0.0%,M: 0.0%,n:130*
Leishmania martiniquensis MHOM/TH/2012/LSCM1	*INSDC*	*GCA_017916325.1*	*2/6/2021*	*C: 100.0%[S: 100.0%,D: 0.0%],F: 0.0%,M: 0.0%,n:130*	*C: 100.0%[S: 100.0%,D: 0.0%],F: 0.0%,M: 0.0%,n:130*
Leishmania orientalis MHOM/TH/2014/LSCM4	*INSDC*	*GCA_017916335.1*	*15/4/2021*	*C: 99.2%[S: 99.2%,D: 0.0%],F: 0.8%,M: 0.0%,n:130*	*C: 100.0%[S: 100.0%,D: 0.0%],F: 0.0%,M: 0.0%,n:130*
Leishmania panamensis strain MHOM/PA/94/PSC-1	*GenBank*	*GCA_000755165.1*	*5/3/2015*	*C: 100.0%[S: 100.0%,D: 0.0%],F: 0.0%,M: 0.0%,n:130*	*C: 100.0%[S: 100.0%,D: 0.0%],F: 0.0%,M: 0.0%,n:130*
Leptomonas pyrrhocoris H10	*GenBank*	*GCA_001293395.1*	*5/5/2015*	*C: 100.0%[S: 99.2%,D: 0.8%],F: 0.0%,M: 0.0%,n:130*	*C: 100.0%[S: 100.0%,D: 0.0%],F: 0.0%,M: 0.0%,n:130*
Leptomonas seymouri ATCC 30220	*GenBank*	*GCA_001299535.1*	*6/5/2015*	*C: 99.2%[S: 99.2%,D: 0.0%],F: 0.8%,M: 0.0%,n:130*	*C: 100.0%[S: 100.0%,D: 0.0%],F: 0.0%,M: 0.0%,n:130*
Leishmania sp. Ghana MHOM/GH/2012/GH5	*INSDC*	*GCA_017918215.1*	*15/4/2021*	*C: 96.9%[S: 96.9%,D: 0.0%],F: 0.0%,M: 3.1%,n:130*	*C: 100.0%[S: 100.0%,D: 0.0%],F: 0.0%,M: 0.0%,n:130*
Leishmania sp. Namibia MPRO/NA/1975/252/LV425	*INSDC*	*GCA_017918225.1*	*15/4/2021*	*C: 99.2%[S: 99.2%,D: 0.0%],F: 0.0%,M: 0.8%,n:130*	*C: 100.0%[S: 100.0%,D: 0.0%],F: 0.0%,M: 0.0%,n:130*
Leishmania tarentolae Parrot-TarII	*CorbeilLab*	*Jun 22, 2011*	*22/6/2011*		
Leishmania tarentolae Parrot Tar II 2019	*INSDC*	*GCA_009731335.1*		*C: 99.2%[S: 98.5%,D: 0.8%],F: 0.0%,M: 0.8%,n:130*	*C: 99.2%[S: 98.5%,D: 0.8%],F: 0.0%,M: 0.8%,n:130*

Leishmania turanica strain LEM423	*GenBank*	*GCA_000441995.1*	*16/12/2014*	*C: 100.0%[S: 100.0%,D: 0.0%],F: 0.0%,M: 0.0%,n:130*	*C: 100.0%[S: 100.0%,D: 0.0%],F: 0.0%,M: 0.0%,n:130*
Paratrypanosoma confusum CUL13	*GenBank*	*GCA_002921335.1*	*2/3/2018*	*C: 96.2%[S: 96.2%,D: 0.0%],F: 2.3%,M: 1.5%,n:130*	*C: 96.9%[S: 96.9%,D: 0.0%],F: 2.3%,M: 0.8%,n:130*
Porcisia hertigi MCOE/PA/1965/C119	*INSDC*	*GCA_017918235.1*	*15/4/2021*	*C: 98.5%[S: 96.9%,D: 1.5%],F: 0.0%,M: 1.5%,n:130*	*C: 100.0%[S: 99.2%,D: 0.8%],F: 0.0%,M: 0.0%,n:130*
Trypanosoma brucei EATRO1125	*INSDC*	*GCA_019096175.1*	*27/8/2021*	*C: 96.2%[S: 96.2%,D: 0.0%],F: 0.0%,M: 3.8%,n:130*	*C: 99.2%[S: 98.5%,D: 0.8%],F: 0.8%,M: 0.0%,n:130*
Trypanosoma brucei Lister strain 427	*GeneDB*	*Jan 16, 2013*	*16/1/2013*		
Trypanosoma brucei Lister strain 427 2018	*GenBank*	*GCA_900497135.1*	*17/12/2018*	*C: 97.7%[S: 97.7%,D: 0.0%],F: 0.0%,M: 2.3%,n:130*	*C: 99.2%[S: 99.2%,D: 0.0%],F: 0.8%,M: 0.0%,n:130*
Trypanosoma congolense IL3000 2019	*GenBank*	*GCA_003013265.1*	*10/9/2019*	*C: 67.7%[S: 66.9%,D: 0.8%],F: 4.6%,M: 27.7%,n:130*	*C: 90.8%[S: 89.2%,D: 1.5%],F: 8.5%,M: 0.8%,n:130*
Trypanosoma cruzi Berenice	*INSDC*	*GCA_013358655.1*	*18/6/2020*	*C: 98.5%[S: 98.5%,D: 0.0%],F: 0.0%,M: 1.5%,n:130*	*C: 98.5%[S: 98.5%,D: 0.0%],F: 0.8%,M: 0.8%,n:130*
Trypanosoma cruzi Brazil A4	*GenBank*	*GCA_015033625.1*	*26/8/2019*	*C: 100.0%[S: 99.2%,D: 0.8%],F: 0.0%,M: 0.0%,n:130*	*C: 99.2%[S: 99.2%,D: 0.0%],F: 0.8%,M: 0.0%,n:130*
Trypanosoma cruzi strain CL	*INSDC*	*GCA_003719155.1*	*5/11/2018*	*C: 86.2%[S: 56.2%,D: 30.0%],F: 10.8%,M: 3.1%,n:130*	*C: 86.2%[S: 56.9%,D: 29.2%],F: 7.7%,M: 6.2%,n:130*
Trypanosoma cruzi CL Brener Esmeraldo-like	*GenBank*	*GCA_000209065.1*	*7/12/2015*	*C: 56.9%[S: 56.2%,D: 0.8%],F: 0.8%,M: 42.3%,n:130*	*C: 60.0%[S: 58.5%,D: 1.5%],F: 2.3%,M: 37.7%,n:130*
Trypanosoma cruzi CL Brener Non-Esmeraldo-like	*GenBank*	*GCA_000209065.1*	*7/12/2015*	*C: 76.9%[S: 76.2%,D: 0.8%],F: 2.3%,M: 20.8%,n:130*	*C: 77.7%[S: 76.9%,D: 0.8%],F: 3.8%,M: 18.5%,n:130*
Trypanosoma cruzi Dm28c 2017	*GenBank*	*GCA_002219105.2*	*15/9/2017*	*C: 100.0%[S: 98.5%,D: 1.5%],F: 0.0%,M: 0.0%,n:130*	*C: 99.2%[S: 97.7%,D: 1.5%],F: 0.8%,M: 0.0%,n:130*
Trypanosoma cruzi Dm28c 2018	*GenBank*	*GCA_003177105.1*	*30/5/2018*	*C: 89.2%[S: 82.3%,D: 6.9%],F: 10.8%,M: 0.0%,n:130*	*C: 93.8%[S: 86.2%,D: 7.7%],F: 6.2%,M: 0.0%,n:130*
Trypanosoma cruzi strain G	*INSDC*	*GCA_003719455.1*	*8/11/2018*	*C: 97.7%[S: 97.7%,D: 0.0%],F: 2.3%,M: 0.0%,n:130*	*C: 97.7%[S: 97.7%,D: 0.0%],F: 2.3%,M: 0.0%,n:130*
Trypanosoma cruzi Sylvio X10/1	*AnderssonLab*	*Mar 18, 2017*	*18/3/2017*		
Trypanosoma cruzi Sylvio X10/1-2012	*GenBank*	*GCA_000188675.2*	*2/10/2012*	*C: 94.6%[S: 94.6%,D: 0.0%],F: 3.1%,M: 2.3%,n:130*	*C: 99.2%[S: 99.2%,D: 0.0%],F: 0.8%,M: 0.0%,n:130*
Trypanosoma cruzi TCC	*GenBank*	*GCA_003177095.1*	*30/5/2018*	*C: 99.2%[S: 17.7%,D: 81.5%],F: 0.0%,M: 0.8%,n:130*	*C: 99.2%[S: 16.2%,D: 83.1%],F: 0.8%,M: 0.0%,n:130*
Trypanosoma cruzi Y C6	*GenBank*	*GCA_015033655.1*	*26/8/2019*	*C: 98.5%[S: 97.7%,D: 0.8%],F: 0.0%,M: 1.5%,n:130*	*C: 98.5%[S: 97.7%,D: 0.8%],F: 0.8%,M: 0.8%,n:130*
Trypanosoma cruzi marinkellei strain B7	*GenBank*	*GCA_000300495.1*	*3/6/2014*	*C: 98.5%[S: 98.5%,D: 0.0%],F: 1.5%,M: 0.0%,n:130*	*C: 99.2%[S: 99.2%,D: 0.0%],F: 0.8%,M: 0.0%,n:130*
Trypanosoma equiperdum OVI	*INSDC*	*GCA_001457755.2*	*5/10/2016*	*C: 94.6%[S: 94.6%,D: 0.0%],F: 0.0%,M: 5.4%,n:130*	*C: 99.2%[S: 99.2%,D: 0.0%],F: 0.8%,M: 0.0%,n:130*
Trypanosoma evansi strain STIB 805	*GenBank*	*GCA_917563935.1*	*3/6/2014*	*C: 95.4%[S: 95.4%,D: 0.0%],F: 0.8%,M: 3.8%,n:130*	*C: 99.2%[S: 99.2%,D: 0.0%],F: 0.8%,M: 0.0%,n:130*
Trypanosoma grayi ANR4	*GenBank*	*GCA_000691245.1*	*17/6/2014*	*C: 96.2%[S: 96.2%,D: 0.0%],F: 3.1%,M: 0.8%,n:130*	*C: 96.9%[S: 96.9%,D: 0.0%],F: 3.1%,M: 0.0%,n:130*

Trypanosoma melophagium St. Kilda	*INSDC*	*GCA_022059095.1*	*8/2/2022*	*C: 100.0%[S: 100.0%,D: 0.0%],F: 0.0%,M: 0.0%,n:130*	*C: 100.0%[S: 100.0%,D: 0.0%],F: 0.0%,M: 0.0%,n:130*
Trypanosoma theileri isolate Edinburgh	*GenBank*	*GCA_002087225.1*	*12/4/2017*	*C: 99.2%[S: 99.2%,D: 0.0%],F: 0.8%,M: 0.0%,n:130*	*C: 99.2%[S: 99.2%,D: 0.0%],F: 0.8%,M: 0.0%,n:130*
