# Supplementary material for: Expanding kinetoplastid genome annotation through protein structure comparison
Source: PLoS Pathog. 2025 Apr 21;21(4):e1013120. doi: 10.1371/journal.ppat.1013120 (PMC12047770; doi:10.1371/journal.ppat.1013120)
Supplement: S2 Table — (PDF) [file ppat.1013120.s006.pdf]

S2 Table. Proteome description of reference organisms used in this work.

Species	Taxonomic lineage	Proteome Id	Protein count (UniProt)	Predicted structures (AFDBv4)	Genome assembly ID	BUSCO
Methanocaldococcus jannaschii	*Archaea, Euryarchaeota, Methanomada group*	*UP000000805*	*1787*	*1773*	*GCA_000091665.1*	*C:98.2%S:98.0%,D:0.2%,F:0.4%,M:1.4%,n:958*
Helicobacter pylori	*Bacteria, Campylobacterota, Epsilonproteobacteria*	*UP000000429*	*1554*	*1538*	*GCA_000008525.1*	*C:99.5%S:99.5%,D:0.0%,F:0.3%,M:0.2%,n:628*
Campylobacter jejuni	*Bacteria, Campylobacterota, Epsilonproteobacteria*	*UP000000799*	*1623*	*1620*	*GCA_000009085.1*	*C:100.0%S:100.0%,D:0.0%,F:0.0%,M:0.0%,n:628*
Neisseria gonorrhoeae	*Bacteria, Pseudomonadota, Betaproteobacteria*	*UP000000535*	*2106*	*2106*	*GCA_000006845.1*	*C:97.6%S:97.6%,D:0.0%,F:0.4%,M:2.0%,n:804*
Klebsiella pneumoniae	*Bacteria, Pseudomonadota, Gammaproteobacteria*	*UP000007841*	*5728*	*5727*	*GCA_000240185.2*	*C:96.6%S:96.1%,D:0.5%,F:1.8%,M:1.6%,n:440*
Haemophilus influenzae	*Bacteria, Pseudomonadota, Gammaproteobacteria*	*UP000000579*	*1704*	*1662*	*GCA_000027305.1*	*C:94.2%S:93.6%,D:0.5%,F:0.9%,M:4.9%,n:1100*
Escherichia coli	*Bacteria, Pseudomonadota, Gammaproteobacteria*	*UP000000625*	*4404*	*4363*	*GCA_000005845.2*	*C:100.0%S:99.1%,D:0.9%,F:0.0%,M:0.0%,n:440*
Shigella dysenteriae	*Bacteria, Pseudomonadota, Gammaproteobacteria*	*UP000002716*	*3897*	*3893*	*GCA_000012005.1*	*C:98.9%S:98.6%,D:0.2%,F:0.0%,M:1.1%,n:440*
Salmonella typhimurium	*Bacteria, Pseudomonadota, Gammaproteobacteria*	*UP000001014*	*4533*	*4526*	*GCA_000006945.2*	*C:100.0%S:99.8%,D:0.2%,F:0.0%,M:0.0%,n:440*
Pseudomonas aeruginosa	*Bacteria, Pseudomonadota, Gammaproteobacteria*	*UP000002438*	*5563*	*5556*	*GCA_000006765.1*	*C:99.5%S:99.0%,D:0.5%,F:0.3%,M:0.3%,n:782*
Mycobacterium tuberculosis	*Bacteria, Terrabacteria group, Actinomycetota*	*UP000001584*	*3995*	*3988*	*GCA_000195955.2*	*C:99.1%S:98.5%,D:0.5%,F:0.4%,M:0.5%,n:743*
Mycobacterium ulcerans	*Bacteria, Terrabacteria group, Actinomycetota*	*UP000020681*	*9033*	*9033*	*GCA_000524035.1*	*C:60.0%S:59.4%,D:0.7%,F:24.9%,M:15.1%,n:743*
Nocardia brasiliensis	*Bacteria, Terrabacteria group, Actinomycetota*	*UP000006304*	*8414*	*8372*	*GCA_000250675.3*	*C:99.6%S:98.1%,D:1.5%,F:0.3%,M:0.1%,n:743*

Mycobacterium leprae	*Bacteria, Terrabacteria group, Actinomycetota*	*UP000000806*	*1603*	*1602*	*GCA_000195855.1*	*C:88.0%S:88.0%,D:0.0%,F:0.5%,M:11.4%,n:743*
Enterococcus faecium	*Bacteria, Terrabacteria group, Bacillota*	*UP000325664*	*2823*	*2823*	*GCA_008728475.1*	*C:100.0%S:99.3%,D:0.7%,F:0.0%,M:0.0%,n:402*
Staphylococcus aureus	*Bacteria, Terrabacteria group, Bacillota*	*UP000008816*	*2889*	*2888*	*GCA_000013425.1*	*C:98.4%S:98.4%,D:0.0%,F:1.3%,M:0.2%,n:450*
Streptococcus pneumoniae	*Bacteria, Terrabacteria group, Bacillota*	*UP000000586*	*2031*	*2030*	*GCA_000007045.1*	*C:100.0%S:100.0%,D:0.0%,F:0.0%,M:0.0%,n:402*
Dictyostelium discoideum	*Eukaryota, Amoebozoa, Evosea*	*UP000002195*	*12726*	*12622*	*GCA_000004695.1*	*C:93.7%S:89.8%,D:3.9%,F:1.6%,M:4.7%,n:255*
Ajellomyces capsulatus	*Eukaryota, Opisthokonta, Fungi*	*UP000001631*	*9214*	*9199*	*GCA_000150115.1*	*C:96.4%S:95.7%,D:0.8%,F:1.7%,M:1.9%,n:4862*
Paracoccidioides lutzii	*Eukaryota, Opisthokonta, Fungi*	*UP000002059*	*8811*	*8794*	*GCA_000150705.2*	*C:97.2%S:97.1%,D:0.1%,F:1.4%,M:1.4%,n:4862*
Cladophialophora carrionii	*Eukaryota, Opisthokonta, Fungi*	*UP000094526*	*11181*	*11170*	*GCA_001700775.1*	*C:96.0%S:95.9%,D:0.1%,F:2.0%,M:2.0%,n:6265*
Sporothrix schenckii	*Eukaryota, Opisthokonta, Fungi*	*UP000018087*	*8673*	*8652*	*GCA_000474925.1*	*C:95.8%S:95.6%,D:0.2%,F:1.1%,M:3.1%,n:3817*
Fonsecaea pedrosoi	*Eukaryota, Opisthokonta, Fungi*	*UP000053029*	*12525*	*12509*	*GCA_000835455.1*	*C:99.3%S:99.1%,D:0.2%,F:0.3%,M:0.4%,n:6265*
Candida albicans	*Eukaryota, Opisthokonta, Fungi*	*UP000000559*	*6036*	*5974*	*GCA_000182965.3*	*C:98.8%S:98.4%,D:0.5%,F:0.7%,M:0.5%,n:2137*
Madurella mycetomatis	*Eukaryota, Opisthokonta, Fungi*	*UP000078237*	*9733*	*9561*	*GCA_001275765.2*	*C:89.3%S:79.5%,D:9.8%,F:0.9%,M:9.8%,n:3817*
Saccharomyces cerevisiae	*Eukaryota, Opisthokonta, Fungi*	*UP000002311*	*6060*	*6039*	*GCA_000146045.2*	*C:99.6%S:97.4%,D:2.2%,F:0.1%,M:0.3%,n:2137*
Schizosaccharomyces pombe	*Eukaryota, Opisthokonta, Fungi*	*UP000002485*	*5117*	*5128*	*GCA_000002945.2*	*C:81.8%S:79.0%,D:2.8%,F:1.4%,M:16.8%,n:1706*
Onchocerca volvulus	*Eukaryota, Opisthokonta, Metazoa*	*UP000024404*	*12225*	*12047*	*GCA_000499405.2*	*C:98.2%S:84.0%,D:14.2%,F:0.5%,M:1.3%,n:3131*
Schistosoma mansoni	*Eukaryota, Opisthokonta, Metazoa*	*UP000008854*	*10770*	*13865*		*C:78.7%S:64.9%,D:13.8%,F:2.6%,M:18.7%,n:954*
Wuchereria bancrofti	*Eukaryota, Opisthokonta, Metazoa*	*UP000270924*	*13000*	*12721*	*GCA_900622535.1*	*C:77.8%S:77.3%,D:0.6%,F:7.1%,M:15.0%,n:3131*
Dracunculus medinensis	*Eukaryota, Opisthokonta, Metazoa*	*UP000274756*	*10868*	*10834*	*GCA_900625125.1*	*C:78.1%S:77.5%,D:0.6%,F:5.8%,M:16.1%,n:3131*
Brugia malayi	*Eukaryota, Opisthokonta, Metazoa*	*UP000006672*	*15168*	*8743*		*C:98.9%S:68.0%,D:30.9%,F:0.2%,M:0.9%,n:3131*
Rattus norvegicus	*Eukaryota, Opisthokonta, Metazoa*	*UP000002494*	*47930*	*21270*	*GCA_015227675.2*	*C:98.1%S:45.6%,D:52.6%,F:0.3%,M:1.6%,n:13798*
Mus musculus	*Eukaryota, Opisthokonta, Metazoa*	*UP000000589*	*54910*	*21615*	*GCA_000001635.9*	*C:99.8%S:51.3%,D:48.5%,F:0.0%,M:0.2%,n:13798*
Homo sapiens	*Eukaryota, Opisthokonta, Metazoa*	*UP000005640*	*82493*	*23391*	*GCA_000001405.29*	*C:99.5%S:37.7%,D:61.8%,F:0.0%,M:0.5%,n:13780*
Drosophila melanogaster	*Eukaryota, Opisthokonta, Metazoa*	*UP000000803*	*22049*	*13458*	*GCA_000001215.4*	*C:100.0%S:41.8%,D:58.2%,F:0.0%,M:0.0%,n:3285*
Danio rerio	*Eukaryota, Opisthokonta, Metazoa*	*UP000000437*	*46578*	*24664*	*GCF_000002035.6*	*C:97.7%S:64.1%,D:33.6%,F:0.6%,M:1.7%,n:3640*

Caenorhabditis elegans	*Eukaryota, Opisthokonta, Metazoa*	*UP000001940*	*26708*	*19694*	*GCA_000002985.3*	*C:100.0%S:74.3%,D:25.7%,F:0.0%,M:0.0%,n:3131*
Strongyloides stercoralis	*Eukaryota, Opisthokonta, Metazoa*	*UP000035681*	*12900*	*12613*	*GCA_029582065.1*	*C:69.9%S:67.1%,D:2.8%,F:0.8%,M:29.4%,n:3131*
Plasmodium falciparum	*Eukaryota, Sar, Alveolata*	*UP000001450*	*5361*	*5187*	*GCA_000002765.3*	*C:99.1%S:98.0%,D:1.1%,F:0.0%,M:0.9%,n:3642*
Zea mays	*Eukaryota, Viridiplantae, Streptophyta*	*UP000007305*	*63256*	*39299*	*GCA_902167145.1*	*C:96.6%S:43.2%,D:53.4%,F:0.8%,M:2.6%,n:4896*
Oryza sativa	*Eukaryota, Viridiplantae, Streptophyta*	*UP000059680*	*48898*	*43649*	*GCA_001433935.1*	*C:84.4%S:78.1%,D:6.3%,F:5.0%,M:10.6%,n:4896*
Glycine max	*Eukaryota, Viridiplantae, Streptophyta*	*UP000008827*	*74862*	*55799*	*GCA_000004515.4*	*C:99.2%S:25.5%,D:73.7%,F:0.2%,M:0.6%,n:5366*
Arabidopsis thaliana	*Eukaryota, Viridiplantae, Streptophyta*	*UP000006548*	*39280*	*27434*	*GCA_000001735.1*	*C:100.0%S:64.3%,D:35.7%,F:0.0%,M:0.0%,n:4596*
Trichuris trichiura	-	*UP000030665*	-	*9564*	-	-
