# Supplementary material for: Expanding kinetoplastid genome annotation through protein structure comparison
Source: PLoS Pathog. 2025 Apr 21;21(4):e1013120. doi: 10.1371/journal.ppat.1013120 (PMC12047770; doi:10.1371/journal.ppat.1013120)
Supplement: S3 Table — (PDF) [file ppat.1013120.s007.pdf]

S3 Table. Gene IDs of kinetoplastid genes in the 4 BUSCO conserved protein clusters with a single SRBH.

Transcription initiation factor TFIID subunit 2 142542at2759	RNA polymerase II subunit A C-terminal domain phosphatase SSU72 1304061at2759	Ubiquinol-cytochrome c chaperone, CBP3 1428265at2759	CTLH, C-terminal LisH motif 1087488at2759
LSCM4_07687	TcIL3000.A.H_000605100	CFAC1_200019900	DQ04_01261000
JKF63_07538	TcIL3000_8_4250	LtaP29.1460	Tb427_040031300
LmxM.12.1250	Tb1125.8.4480	Tc_MARK_3098	TEOVI_000465600
LTRL590_120014700	Tb927.8.4480	ECC02_007338	TevSTIB805.4.3010
LMJFC_120019600	Tb427_080049700	LSM04_000858	Tbg972.4.2870
LdCL_120020400	TevSTIB805.8.4620	LPMP_291340	Tb927.4.2890
LARLEM1108_120014700	Tbg972.8.4240	TcCL_Unassigned05796	Tb1125.4.2890
LGELEM452_120015700	TEOVI_000095600	Tbg972.3.4250	Tb427.04.2890
LINF_120016700	Tb427.08.4480	LINF_290018900	C3747_25g139
LtaP12.1060	TcCL_ESM06005	TcBrA4_0128780	BCY84_19026
LmjF.12.1250	TcBrA4_0019340	CUR178_04195	TcCL_ESM08092
LTULEM423_120014800	TRSC58_05615	PCON_0043550	TcG_01971
LtaPh_1210600	TcCLB.508989.70	LbrM.29.1380	TcCLB.504253.20
LMJLV39_120014700	C3747_125g26	DQ04_00341020	C4B63_20g20
LdBPK_120830.1	TcCL_NonESM13541	TEOVI_000029700	TcYC6_0088390
LAEL147_000172000	TcG_02193	LAMA_000114800	TcBrA4_0122600
LAMA_000192700	BCY84_05375	LAEL147_000528700	ECC02_007096
LBRM2903_200076400	C3747_66g56	Tb1125.3.3890	TcCL_NonESM02399
LPMP_120930	Tc_MARK_9824	TRSC58_06831	TCSYLVIO_003916
LbrM.12.2.000930	ECC02_003063	TM35_000092450	TCSYLVIO_003917
LbrM.12.0930	TcCLB.509569.160	BCY84_15100	TcCLB.507735.60
LPAL13_120014300	C4B63_21g49	JKF63_00542	C3747_225g7
LpyrH10_23_0150	TCDM_03304	TvY486_0303210	Tc_MARK_2612

Lsey_0082_0120	TcYC6_0064470	BSAL_59245	LSM04_001868
LMARLEM2494_120014900	TCSYLVIO_000479	LpyrH10_08_1640	TvY486_0402750
LSCM1_07901	DQ04_03141080	Baya_040_0230	TM35_000014310
CFAC1_010017900	TvY486_0803950	LENLEM3045_290019700	
EMOLV88_120015600	LSM04_006502	TcG_02892	
	TM35_000252160	LGELEM452_290019500	
		Tb927.3.3890	
		TcCL_NonESM06154	
		LMARLEM2494_290019200	
		C3747_13g293	
		TcIL3000_3_2500	
		LdCL_290019000	
		TRSC58_02287	
		TCSYLVIO_004299	
		TcYC6_0109600	
		LtaPh_2914600	
		LMJFC_290020700	
		TevSTIB805.3.4110	
		LSCM4_03585	
		LdBPK_291390.1	
		LBRM2903_290020300	
		LmjF.29.1300	
		LDHU3_29.1890	
		LMJLV39_290019400	
		EMOLV88_360060800	
		TcIL3000.A.H_000341700	
		Tb427.03.3890	
		LSCM1_04426	

LTRL590_290020000
C3747_47g274
TcCLB.509999.80
TCDM_04301
LARLEM1108_290019500
LdBPK.29.2.001390
Tb427_030041700
LTULEM423_290019200
Lsey_0083_0040
LPAL13_290017700
C4B63_10g146
LmxM.08_29.1300
