# Supplementary material for: Expanding kinetoplastid genome annotation through protein structure comparison
Source: PLoS Pathog. 2025 Apr 21;21(4):e1013120. doi: 10.1371/journal.ppat.1013120 (PMC12047770; doi:10.1371/journal.ppat.1013120)
Supplement: S4 Table — (PDF) [file ppat.1013120.s008.pdf]

S4 Table. Gene IDs of kinetoplastid genes inside the 5 BUSCO conserved protein clusters with multiple SRBH.

Golgi to ER traffic protein 4 1030907at2759	Co-chaperone Hsc20 1129824at2759	TFIIH subunit Tfb4/GTF2H3 1220881at2759	tRNA (guanine-N(7)-)-methyltransferase non-catalytic subunit 937275at2759	Tetratricopeptide-like helical domain superfamily 331411at2759
LbrM.13.2.001300	LSM04_005221	TRSC58_02409	Tc_MARK_3604	LbrM.32.2.004190
LPAL13_200009800	TM35_000111840	TCDM_05800	C3747_40g223	LAEL147_000663700
LAEL147_000708700	TEOVI_000541400	TcBrA4_0035310	BCY84_10939	LMJLV39_320047400
LmxM.33.0540	Tb427.03.1760	TcYC6_0050670	TCSYLVIO_004859	LTRL590_320047700
LMJLV39_340011600	Tb927.3.1760	BCY84_08330	TcCL_NonESM09808	LdCL_320047000
LAMA_000734700	TevSTIB805.3.1770	Tc_MARK_3393	TcYC6_0124730	LtaP32.4120
LMARLEM2494_340011200	Tb1125.3.1760	TcCLB.509073.60	TCDM_10622	LMJFC_320053000
LBRM2903_200011400	Tbg972.3.1610	TcCLB.508707.149	TcG_06461	LAMA_000694300
LINF_340011000	TRSC58_02379	C3747_234g54	BCY84_06378	LmjF.32.3950
LdBPK.34.2.000560	TCSYLVIO_006355	TcCL_NonESM05049	TcBrA4_0015470	LbrM.32.4190
LDHU3_34.0950	TcYC6_0119320	ECC02_004028	C4B63_84g58	LGELEM452_320047800
LtaP34.0600	TcCLB.510091.50	TcCL_ESM05086	ECC02_001902	JKF63_02758
LGELEM452_340011000	BCY84_03923	C4B63_80g28	TcCL_ESM11043	LmxM.31.3950
LSCM4_00818	TcSYL_0019370	C3747_68g50	C3747_60g161	CUR178_02533
LdBPK_340560.1	BCY84_13531	TcG_04138	TcCLB.509639.20	LSCM4_03084
LtaPh_3406000	C3747_20g82	TCSYLVIO_005377	TcCLB.507711.120	LARLEM1108_320047800
LENLEM3045_340010900	C3747_45g247	CFAC1_190042600	TRSC58_00287	LINF_320046700
LdCL_340011200	TcBrA4_0082700	LtaP32.3070	DQ04_00121260	LPMP_324080
CUR178_01385	TCDM_03945	LbrM.32.2.003130	TcIL3000.A.H_000817700	LDHU3_32.5270
LPMP_200550	TcCL_NonESM05362	LGELEM452_320036700	TcIL3000_10_10140	LMARLEM2494_320048200
LMJFC_340011900	ECC02_002253	LBRM2903_320039200	TcIL3000.A.H_000824600	LSCM1_02053
LARLEM1108_340011500	Tc_MARK_5057	LAMA_000684000	Tb427_100119200	LtaPh_3241200
LbrM.13.1300	TcCL_ESM04768	LMARLEM2494_320036900	Tb427.10.11210	LTULEM423_320048400
EMOLV88_340010500	TcG_06056	LbrM.32.3130	TEOVI_000133900	LPAL13_320048100

LmjF.34.0540	TcCLB.510421.300	CUR178_02424	TevSTIB805.10.11780	LENLEM3045_320047900
LTULEM423_340011000	C4B63_11g76	LARLEM1108_320036700	Tb1125.10.11210	LdBPK_324100.1
LTRL590_340011100	DQ04_01111060	LDHU3_32.3810	Tbg972.10.13550	LdBPK.32.2.004100
LmjF.34.0540	TvY486_0301062	LSCM4_02974	Tb927.10.11210	EMOLV88_320043300
CFAC1_290034100	JKF63_04675	LPAL13_320036700		Lsey_0261_0050
JKF63_01649	Lsey_0012_0480	LINF_320036000		CFAC1_300048100
Lsey_0144_0120	CFAC1_230016200	LmxM.31.2885		LpyrH10_02_4410
LpyrH10_05_0810	Baya_095_0230	LTULEM423_320037300		C4B63_27g161
C3747_57g63	LmxM.25.1690	LSCM1_01941		Tc_MARK_5200
TcYC6_0126640	LtaP25.1770	LENLEM3045_320037200		TcG_09286
TcBrA4_0017580	LtaPh_2517700	LmjF.32.2885		TcCLB.504005.50
TcCLB.510187.140	LAMA_000495100	LPMP_323030		LSM04_008793
TCDM_06586	LBRM2903_250025800	LtaPh_3230700		TcYC6_0035890
BCY84_22873	LdBPK.25.2.001760	LAEL147_000652600		TcCL_ESM12074
C4B63_81g55	LbrM.25.2310	LdBPK_323030.1		BCY84_01300
Tc_MARK_6187	LdBPK_251760.1	LMJFC_320039400		TCSYLVIO_006462
C3747_114g44	LSCM1_05385	LMJLV39_320036400		ECC02_006056
TCSYLVIO_005008	LARLEM1108_250023400	LTRL590_320036700		C3747_107g90
ECC02_002003	LPMP_251760	LdCL_320036300		TcBrA4_0109500
	LPAL13_000047100	LdBPK.32.2.003030		C3747_74g56
	LTRL590_250024000	JKF63_02652		TM35_000312400
	LMJFC_250027500	LpyrH10_02_3320		DQ04_03381070
	LAEL147_000417800	Lsey_0010_0360		TCDM_14112
	LSCM4_04526			Tbg972.11.13490
	LDHU3_25.2200			Tb927.11.12050
	LENLEM3045_250024000			Tb427tmp.01.3870
	LMARLEM2494_250023400			Tb427_110137300
	EMOLV88_250022800			Tb1125.11.12050

LdCL_250023300	TEOVI_000908500
LMJLV39_250024300	TcIL3000.11.12680
LmjF.25.1690	TevSTIB805.11_01.12430
LTULEM423_250024100	
LGELEM452_250023900	
LINF_250023500	
CUR178_05280	
LbrM.25.2.002310	
LpyrH10_10_2160	
LPAL13_330036000	
LMJFC_330040500	
LMARLEM2494_330034800	
LSCM1_02777	
LBRM2903_330037600	
LGELEM452_330036400	
LINF_330036200	
LmjF.33.2690	
EMOLV88_330032700	
LDHU3_33.3970	
LARLEM1108_330033000	
LdBPK.33.2.002830	
LmxM.32.2690	
LMJLV39_330038000	
LPMP_332810	
JKF63_02300	
LENLEM3045_330035800	
LtaP33.2920	
LTULEM423_330036100	

LSCM4_02597
LdBPK_332830.1
LdCL_330035400
LbrM.33.2970
LAEL147_000695400
CUR178_02045
LbrM.33.2.002970
LTRL590_330035600
LAMA_000723200
LtaPh_3329200
CFAC1_210037700
Lsey_0151_0010
LpyrH10_03_4980
