# Supplementary material for: Expanding kinetoplastid genome annotation through protein structure comparison
Source: PLoS Pathog. 2025 Apr 21;21(4):e1013120. doi: 10.1371/journal.ppat.1013120 (PMC12047770; doi:10.1371/journal.ppat.1013120)
Supplement: S5 Table — (PDF) [file ppat.1013120.s009.pdf]

S5 Table. Detailed information of results for "case study" SRBH.

Query uniport accession (kinetoplastid)	Organism in clusters	Cluster representative	BUSCO ID of model organism hit	BUSCO Description	Model Organism	Protein names (model organism)	Target uniport accession (Model Organism)	GO description TrypTagg	Sequence identitiy of structural aligment
**A0A640KRB8**	Leishmania: 26, Leptomonas: 2, Crithidia: 1, Endotrypanum: 1, Porcisia: 1	LbrM.13.2.001300	1030907at2759	Golgi to ER traffic protein 4	AJECG, ,PARBA, ,SPOS1	DUF410 domain-containing protein	C0P182, C1GXR3, U7PSW4		0.111, 0.112, 0.091
**A0A2V2WCY5**	Trypanosoma: 10	C3747_57g63	1030907at2759	Golgi to ER traffic protein 4	BRUMA,CANAL,DICDI,PLAF7	BMA-CEE-1,Golgi to ER traffic protein 4, Golgi to ER traffic protein 4 homolog,Uncharacterized protein	A0A0K0K0X7,A0A1D8PND7,Q54TH4,Q8IL82		0.114, 0.133, 0.124, 0.122
**Q4D4T7**	Trypanosoma: 23	DQ04_01261000	1087488at2759	CTLH, C-terminal LisH motif	MOUSE,RAT,HUMAN	E3 ubiquitin-protein transferase MAEA (EC 2.3.2.27) (Erythroblast macrophage protein) (Macrophage erythroblast attacher), E3 ubiquitin-protein transferase MAEA (EC 2.3.2.27) (Macrophage erythroblast attacher), E3 ubiquitin-protein transferase MAEA (EC 2.3.2.27) (Cell proliferation-inducing gene 5 protein) (Erythroblast macrophage protein) (Human lung cancer oncogene 10 protein) (HLC-10) (Macrophage erythroblast attacher) (P44EMLP)	Q4VC33,Q5RKJ1,Q7L5Y9	cytoplasm(weak; points)	0.142, 0.139
**Q57ZD5**	Trypanosoma: 25	LSM04_005221	1129824at2759	Co-chaperone Hsc20	ORYSJ	Co-chaperone Hsc20 family protein, expressed (Os12g0456200 protein) (cDNA clone:J023020P09, full insert sequence)	Q2QRM4	cytoplasm(points)	0.168
**Q4Q9S1**	Leishmania: 27, Leptomonas: 2, Blechomonas: 1, Crithidia: 1, Endotrypanum: 1, Porcisia: 1	JKF63_04675	1129824at2759	Co-chaperone Hsc20	DROME,RAT,HUMAN,ARATH	Heat shock protein cognate 20 (MIP14027p), HscB mitochondrial iron-sulfur cluster co-chaperone, Iron-sulfur cluster co-chaperone protein HscB (DnaJ	A8JNT7,D3ZME7,Q8IWL3,Q8L7K4		0.215, 0.212, 0.226, 0.142

						homolog subfamily C member 20) Cleaved into: Iron-sulfur cluster co-chaperone protein HscB, cytoplasmic (C-HSC20); Iron-sulfur cluster co-chaperone protein HscB, mitochondrial, Iron-sulfur cluster co-chaperone protein HscB homolog (AtHscB)			
**A0A640KWU3**	Leishmania: 27, Leptomonas: 2, Crithidia: 1, Endotrypanum: 1, Porcisia: 1	LPAL13_330036000	1129824at2759	Co-chaperone Hsc20	AJECG,PARBA	DnaJ domain-containing protein,J-type co-chaperone JAC1	C0NFZ6,C1H2E3		0.128, 0.149
**Q4E262**	Trypanosoma: 14	TRSC58_02409	1220881at2759	TFIIH subunit Tfb4/GTF2H3	SOYBN,SCHPO,YEAST	General transcription and DNA repair factor IIH subunit TFB4 (RNA polymerase II transcription factor B subunit 4), General transcription and DNA repair factor IIH subunit tfb4 (TFIIH subunit tfb4) (RNA polymerase II transcription factor B subunit 4), General transcription and DNA repair factor IIH subunit TFB4 (TFIIH subunit TFB4) (RNA polymerase II transcription factor B 34 kDa subunit) (RNA polymerase II transcription factor B p34 subunit) (RNA polymerase II transcription factor B subunit 4)	A0A0R0JLP0,O74366,Q12004		0.152, 0.15, 0.091
**A0A0N1IME3**	Leishmania: 27, Leptomonas: 2, Crithidia: 1, Porcisia: 1	CFAC1_190042600	1220881at2759	TFIIH subunit Tfb4/GTF2H3	AJECG	General transcription and DNA repair factor IIH subunit TFB4 (TFIIH subunit TFB4) (RNA polymerase II transcription factor B subunit 4)	C0NNU2		0.071
**Q4DKS2**	Trypanosoma: 27	TcIL3000.A.H_000605100	1304061at2759	RNA polymerase II subunit A C-terminal domain phosphatase SSU72	ARATH,DROME	RNA polymerase II subunit A C-terminal domain phosphatase SSU72 (CTD phosphatase SSU72) (EC 3.1.3.16)	A0A1P8AMK1,Q9VWE4	nucleoplasm	0.117, 0.105
**E9AGI7**	Leishmania: 23, Leptomonas: 2, Crithidia: 1,	LSCM4_07687	142542at2759	Transcription initiation factor TFIID subunit 2	9EURO2,9EURO1,CANAL,PARBA,RAT	Transcription initiation factor TFIID subunit 2, Transcription initiation	A0A0D2DTU3,A0A1C1CSD0,A0A1D8PQF6,C1H2G2,F1LNY6		0.113, 0.124, 0.122,

	Endotrypanum: 1, Porcisia: 1					factor TFIID subunit 2 (Transcription initiation factor TFIID 150 kDa subunit)		0.115, 0.099
**A4I4I7**	Trypanosoma: 26, Leishmania: 26, Leptomonas: 2, Bodo: 1, Blechomonas: 1, Crithidia: 1, Endotrypanum: 1, Porcisia: 1, Paratrypanosoma: 1	CFAC1_200019900	1428265at2759	Ubiquinol-cytochrome c chaperone, CBP3	SOYBN,YEAST,CANAL,ORYSJ	Ubiquinol-cytochrome c chaperone domain-containing protein, Protein CBP3, mitochondrial Os07g0490300 protein (cDNA clone:J013149P03, full insert sequence)	I1MDZ1,P21560,Q5AC35,Q7XHR1	0.134, 0.126, 0.131, 0.123
**A4I8L3**	Leishmania: 26, Leptomonas: 2, Crithidia: 1, Endotrypanum: 1, Porcisia: 1	LbrM.32.2.004190	331411at2759	Tetratricopeptide-like helical domain superfamily	DANRE,SCHPO,HUMAN,CAEEL,MOUSE	Cleavage stimulation factor subunit 3 (Cleavage stimulation factor, 3 pre-RNA, subunit 3), mRNA 3-end-processing protein rna14" Cleavage stimulation factor subunit 3 (CF-1 77 kDa subunit) (Cleavage stimulation factor 77 kDa subunit) (CSTF 77 kDa subunit) (CstF-77) Suppressor of forked domain-containing protein	F1QIB2,O14233,Q12996,Q19866,Q99LI7	0.14, 0.097, 0.123, 0.146, 0.124
**Q4CY43**	Trypanosoma: 23	C4B63_27g161	331411at2759	Tetratricopeptide-like helical domain superfamily	DRAME,WUCBA,SCHMA,RAT	Suf domain-containing protein, Suppressor of forked domain-containing protein, Putative cleavage stimulation factor, Cleavage stimulation factor subunit 3	A0A158Q688,A0A3P7DR84,A0A3Q0KPI3,F1M4W7	0.135, 0.133, 0.139, 0.142
**Q4DZR6**	Trypanosoma: 15	Tc_MARK_3604	937275at2759	tRNA (guanine-N(7)-)-methyltransferase non-catalytic subunit	DANRE,SCHPO,YEAST,MOUSE	tRNA (guanine-N(7)-)-methyltransferase non-catalytic subunit wdr4 (WD repeat-containing protein 4), tRNA (guanine-N(7)-)-methyltransferase non-catalytic subunit trm82 (Transfer RNA methyltransferase 82), tRNA (guanine-N(7)-)-methyltransferase non-catalytic subunit TRM82 (Transfer RNA methyltransferase 82), tRNA (guanine-N(7)-)-methyltransferase non-catalytic subunit , WDR4 (Protein Wuho homolog) (mWH) (WD	A4IGH4,O74863,Q03774,Q9EP82	0.17, 0.156, 0.149, 0.139

							repeat-containing protein 4)				
**G0UXW8**	Trypanosoma: 9	TcIL3000.A.H_000817700	937275at2759	tRNA (guanine-N(7)-)-methyltransferase non-catalytic subunit	CANAL		tRNA (guanine-N(7)-)-methyltransferase non-catalytic subunit TRM82 (Transfer RNA methyltransferase 82)	Q5AH60			0.137
