# Supplementary material for: Expanding kinetoplastid genome annotation through protein structure comparison
Source: PLoS Pathog. 2025 Apr 21;21(4):e1013120. doi: 10.1371/journal.ppat.1013120 (PMC12047770; doi:10.1371/journal.ppat.1013120)
Supplement: S6 Table — (PDF) [file ppat.1013120.s010.pdf]

S6 Table Protein Domains Identified by InterProScan. This table summarizes the domain information for all members of each case study cluster. Columns ending in "_query" list the unique domains identified by InterProScan (each domain is counted once, even if repeated, and domains found in only one member are included). Columns ending in "_RefOrg" show the top-hit data for that cluster from the reference organisms in our database.

cluster_representer	*databases_domain_code_query*	*interprot_code_query*	*databases_domain_description_query*	*UniProt_ID_RefOrg*	*databases_domain_code_RefOrg*	*interprot_code_RefOrg*	*databases_domain_description_RefOrg*
TcCLB.509569.160	No domain detected	No domain detected	No domain detected	A0A1D6MYY7	PF04722, PTHR20383	IPR006811	Ssu72-like protein, RNA POLYMERASE II SUBUNIT A C-TERMINAL DOMAIN PHOSPHATASE
TcCLB.509569.160	No domain detected	No domain detected	No domain detected	A0A1P8AMK1	PTHR20383, PF04722	IPR006811	RNA POLYMERASE II SUBUNIT A C-TERMINAL DOMAIN PHOSPHATASE, Ssu72-like protein
TcCLB.509569.160	No domain detected	No domain detected	No domain detected	A0A1W2PQ27	PTHR20383, PF04722	IPR006811	RNA POLYMERASE II SUBUNIT A C-TERMINAL DOMAIN PHOSPHATASE, Ssu72-like protein
TcCLB.509569.160	No domain detected	No domain detected	No domain detected	A0A1W2PQC6	PF04722, PTHR20383	IPR006811	Ssu72-like protein, RNA POLYMERASE II SUBUNIT A C-TERMINAL DOMAIN PHOSPHATASE
TcCLB.509569.160	No domain detected	No domain detected	No domain detected	Q9VWE4	PF04722, PTHR20383	IPR006811	Ssu72-like protein, RNA POLYMERASE II SUBUNIT A C-TERMINAL DOMAIN PHOSPHATASE

TcCLB.508707.149	No domain detected	No domain detected	No domain detected	A0A0R0JLP0	PTHR12831, G3DSA:3.40.50.410, PF03850	IPR004600, IPR036465	TRANSCRIPTION INITIATION FACTOR IIH TFIIH , POLYPEPTIDE 3-RELATED, von Willebrand factor, type A domain, Transcription factor Tfb4
TcCLB.508707.149	No domain detected	No domain detected	No domain detected	A0A5K4F918	PTHR12831, PF03850, G3DSA:3.40.50.410	IPR004600, IPR036465	TRANSCRIPTION INITIATION FACTOR IIH TFIIH , POLYPEPTIDE 3-RELATED, Transcription factor Tfb4, von Willebrand factor, type A domain
Tb927.3.1760	PTHR14021, G3DSA:1.10.287.110, SSF46565, SSF47144, G3DSA:1.20.1280.20, cd06257	IPR004640, IPR036869, IPR036386, IPR001623	IRON-SULFUR CLUSTER CO-CHAPERONE PROTEIN HSCB, DnaJ domain, Chaperone J-domain, HSC20 (HSCB), C-terminal oligomerisation domain, -, DnaJ	A0A3P7EC88	G3DSA:1.20.1280.20, SSF46565, PF07743, G3DSA:1.10.287.110, cd06257, G3DSA:3.30.70.330, SSF47144, TIGR00714, PTHR14021	IPR036386, IPR036869, IPR009073, IPR001623, IPR012677, IPR004640	-, Chaperone J-domain, HSCB C-terminal oligomerisation domain, DnaJ domain, DnaJ, HSC20 (HSCB), C-terminal oligomerisation domain, Fe-S protein assembly co-chaperone HscB, IRON-SULFUR CLUSTER CO-CHAPERONE PROTEIN HSCB
Tb927.3.1760	PTHR14021, G3DSA:1.10.287.110, SSF46565, SSF47144,	IPR004640, IPR036869, IPR036386, IPR001623	IRON-SULFUR CLUSTER CO-CHAPERONE PROTEIN HSCB, DnaJ domain, Chaperone J-domain, HSC20	A8JNT7	G3DSA:1.20.1280.20, G3DSA:1.10.287.110, PF07743, SSF47144,	IPR036386, IPR036869, IPR009073, IPR004640, IPR001623	-, DnaJ domain, HSCB C-terminal oligomerisation domain, HSC20 (HSCB), C-terminal oligomerisation domain,

	G3DSA:1.20.1280.20, cd06257		(HSCB), C-terminal oligomerisation domain, -, DnaJ		TIGR00714, cd06257, SSF46565, PTHR14021		Fe-S protein assembly co-chaperone HscB, DnaJ, Chaperone J-domain, IRON-SULFUR CLUSTER CO-CHAPERONE PROTEIN HSCB
Tb927.3.1760	PTHR14021, G3DSA:1.10.287.110, SSF46565, SSF47144, G3DSA:1.20.1280.20, cd06257	IPR004640, IPR036869, IPR036386, IPR001623	IRON-SULFUR CLUSTER CO-CHAPERONE PROTEIN HSCB, DnaJ domain, Chaperone J-domain, HSC20 (HSCB), C-terminal oligomerisation domain, -, DnaJ	P0A6L9	PF07743, PTHR14021, G3DSA:1.20.1280.20, TIGR00714, G3DSA:1.10.287.110, SM00271, SSF47144, cd06257, SSF46565	IPR009073, IPR004640, IPR036386, IPR036869, IPR001623	HSCB C-terminal oligomerisation domain, IRON-SULFUR CLUSTER CO-CHAPERONE PROTEIN HSCB, -, Fe-S protein assembly co-chaperone HscB, DnaJ domain, dnaj_3, HSC20 (HSCB), C-terminal oligomerisation domain, DnaJ, Chaperone J-domain
Tb927.3.1760	PTHR14021, G3DSA:1.10.287.110, SSF46565, SSF47144, G3DSA:1.20.1280.20, cd06257	IPR004640, IPR036869, IPR036386, IPR001623	IRON-SULFUR CLUSTER CO-CHAPERONE PROTEIN HSCB, DnaJ domain, Chaperone J-domain, HSC20 (HSCB), C-terminal oligomerisation domain, -, DnaJ	Q8IWL3	SSF46565, PF07743, PF18256, SSF47144, G3DSA:1.10.287.110, TIGR00714, PTHR14021, G3DSA:1.20.1280.20	IPR036869, IPR009073, IPR040682, IPR036386, IPR004640	Chaperone J-domain, HSCB C-terminal oligomerisation domain, Co-chaperone HscB tetracysteine metal binding motif, HSC20 (HSCB), C-terminal oligomerisation domain, DnaJ domain, Fe-S protein assembly co-chaperone HscB, IRON-SULFUR CLUSTER CO-

							CHAPERONE PROTEIN HSCB, -
Tb927.3.1760	PTHR14021, G3DSA:1.10.287.110, SSF46565, SSF47144, G3DSA:1.20.1280.20, cd06257	IPR004640, IPR036869, IPR036386, IPR001623	IRON-SULFUR CLUSTER CO-CHAPERONE PROTEIN HSCB, DnaJ domain, Chaperone J-domain, HSC20 (HSCB), C-terminal oligomerisation domain, -, DnaJ	Q8K3A0	SSF46565, SSF47144, G3DSA:1.10.287.110, G3DSA:1.20.1280.20, PTHR14021, PF07743, PF18256, TIGR00714	IPR036869, IPR036386, IPR004640, IPR009073, IPR040682	Chaperone J-domain, HSC20 (HSCB), C-terminal oligomerisation domain, DnaJ domain, -, IRON-SULFUR CLUSTER CO-CHAPERONE PROTEIN HSCB, HSCB C-terminal oligomerisation domain, Co-chaperone HscB tetracysteine metal binding motif, Fe-S protein assembly co-chaperone HscB
Tb927.3.1760	PTHR14021, G3DSA:1.10.287.110, SSF46565, SSF47144, G3DSA:1.20.1280.20, cd06257	IPR004640, IPR036869, IPR036386, IPR001623	IRON-SULFUR CLUSTER CO-CHAPERONE PROTEIN HSCB, DnaJ domain, Chaperone J-domain, HSC20 (HSCB), C-terminal oligomerisation domain, -, DnaJ	Q8ZN41	SSF47144, TIGR00714, PF07743, SSF46565, G3DSA:1.10.287.110, SM00271, G3DSA:1.20.1280.20, PTHR14021	IPR036386, IPR004640, IPR009073, IPR036869, IPR001623	HSC20 (HSCB), C-terminal oligomerisation domain, Fe-S protein assembly co-chaperone HscB, HSCB C-terminal oligomerisation domain, Chaperone J-domain, DnaJ domain, dnaj_3, -, IRON-SULFUR CLUSTER CO-CHAPERONE PROTEIN HSCB
TcCLB.504005.50	G3DSA:1.25.40.10, SSF48452, PTHR19980	IPR011990, IPR045243	Tetratricopeptide repeat domain, TPR-like, RNA CLEAVAGE STIMULATION FACTOR	A0A1D6M355	PTHR19980, SSF48452, PF05843	IPR045243, IPR011990, IPR008847	RNA CLEAVAGE STIMULATION FACTOR, TPR-like, Suppressor of forked protein (Suf)

TcCLB.504253.20	PTHR23353, PTHR12170, No domain detected	IPR053019, IPR045098, No domain detected	RAB-GAP/TBC-RELATED, MACROPHAGE ERYTHROBLAST ATTACHER-RELATED, No domain detected	Q4VC33	PTHR12170, PF10607, SM00757, SM00667, SM00668	IPR045098, IPR024964, IPR013144, IPR006594, IPR006595	MACROPHAGE ERYTHROBLAST ATTACHER-RELATED, CTLH/CRA C-terminal to LisH motif domain, toby_final6, Lish, ctlh
TcCLB.504253.20	PTHR23353, PTHR12170, No domain detected	IPR053019, IPR045098, No domain detected	RAB-GAP/TBC-RELATED, MACROPHAGE ERYTHROBLAST ATTACHER-RELATED, No domain detected	Q5RKJ1	PTHR12170, SM00757, PF10607, SM00668, SM00667	IPR045098, IPR013144, IPR024964, IPR006595, IPR006594	MACROPHAGE ERYTHROBLAST ATTACHER-RELATED, toby_final6, CTLH/CRA C-terminal to LisH motif domain, ctlh, Lish
TcCLB.504253.20	PTHR23353, PTHR12170, No domain detected	IPR053019, IPR045098, No domain detected	RAB-GAP/TBC-RELATED, MACROPHAGE ERYTHROBLAST ATTACHER-RELATED, No domain detected	Q7L5Y9	SM00667, PTHR12170, PF10607, SM00757, SM00668	IPR006594, IPR045098, IPR024964, IPR013144, IPR006595	Lish, MACROPHAGE ERYTHROBLAST ATTACHER-RELATED, CTLH/CRA C-terminal to LisH motif domain, toby_final6, ctlh
LmjF.25.1690	PTHR14021, G3DSA:1.10.287.110, SSF46565	IPR004640, IPR036869	IRON-SULFUR CLUSTER CO-CHAPERONE PROTEIN HSCB, DnaJ domain, Chaperone J-domain	A0A077Z217	TIGR00714, G3DSA:1.20.1280.20, SSF46565, G3DSA:1.10.287.110, PTHR14021, cd06257, SM00271, PF07743, SSF47144	IPR004640, IPR036386, IPR036869, IPR001623, IPR009073	Fe-S protein assembly co-chaperone HscB, -, Chaperone J-domain, DnaJ domain, IRON-SULFUR CLUSTER CO-CHAPERONE PROTEIN HSCB, DnaJ, dnaj_3, HSCB C-terminal oligomerisation domain, HSC20 (HSCB), C-terminal oligomerisation domain

LmjF.25.1690	PTHR14021, G3DSA:1.10.287.110, SSF46565	IPR004640, IPR036869	IRON-SULFUR CLUSTER CO-CHAPERONE PROTEIN HSCB, DnaJ domain, Chaperone J-domain	A0A0K0JGP1	cd06257, TIGR00714, G3DSA:1.10.287.110, PF07743, SSF47144, PTHR14021, SSF46565, G3DSA:1.20.1280.20	IPR001623, IPR004640, IPR036869, IPR009073, IPR036386	DnaJ, Fe-S protein assembly co-chaperone HscB, DnaJ domain, HSCB C-terminal oligomerisation domain, HSC20 (HSCB), C-terminal oligomerisation domain, IRON-SULFUR CLUSTER CO-CHAPERONE PROTEIN HSCB, Chaperone J-domain, -
LmjF.25.1690	PTHR14021, G3DSA:1.10.287.110, SSF46565	IPR004640, IPR036869	IRON-SULFUR CLUSTER CO-CHAPERONE PROTEIN HSCB, DnaJ domain, Chaperone J-domain	D3ZME7	PF18256, G3DSA:1.10.287.110, PTHR14021, G3DSA:1.20.1280.20, SSF47144, PF07743, TIGR00714, SSF46565	IPR040682, IPR036869, IPR004640, IPR036386, IPR009073	Co-chaperone HscB tetracysteine metal binding motif, DnaJ domain, IRON-SULFUR CLUSTER CO-CHAPERONE PROTEIN HSCB, -, HSC20 (HSCB), C-terminal oligomerisation domain, HSCB C-terminal oligomerisation domain, Fe-S protein assembly co-chaperone HscB, Chaperone J-domain
LmjF.25.1690	PTHR14021, G3DSA:1.10.287.110, SSF46565	IPR004640, IPR036869	IRON-SULFUR CLUSTER CO-CHAPERONE PROTEIN HSCB, DnaJ domain, Chaperone J-domain	Q8K3A0	SSF46565, SSF47144, PF18256, TIGR00714, G3DSA:1.20.1280.2	IPR036869, IPR036386, IPR040682, IPR004640, IPR009073	Chaperone J-domain, HSC20 (HSCB), C-terminal oligomerisation domain, Co-chaperone HscB tetracysteine

					0, PTHR14021, G3DSA:1.10.287.110, PF07743		metal binding motif, Fe-S protein assembly co-chaperone HscB, -, IRON-SULFUR CLUSTER CO-CHAPERONE PROTEIN HSCB, DnaJ domain, HSCB C-terminal oligomerisation domain
LmjF.25.1690	PTHR14021, G3DSA:1.10.287.110, SSF46565	IPR004640, IPR036869	IRON-SULFUR CLUSTER CO-CHAPERONE PROTEIN HSCB, DnaJ domain, Chaperone J-domain	Q9HXJ1	SSF47144, G3DSA:1.10.287.110, PF07743, G3DSA:1.20.1280.20, SSF46565, PTHR14021, cd06257, TIGR00714, SM00271	IPR036386, IPR036869, IPR009073, IPR004640, IPR001623	HSC20 (HSCB), C-terminal oligomerisation domain, DnaJ domain, HSCB C-terminal oligomerisation domain, -, Chaperone J-domain, IRON-SULFUR CLUSTER CO-CHAPERONE PROTEIN HSCB, DnaJ, Fe-S protein assembly co-chaperone HscB, dnaj_3
LtaPh_3329200	SSF46565, G3DSA:1.10.287.110, PTHR43908, cd06257, PTHR24074, SM00271, PF00226, PR00625	IPR036869, IPR051100, IPR001623, IPR050817	Chaperone J-domain, DnaJ domain, AT29763P-RELATED, DnaJ, CO-CHAPERONE PROTEIN DJLA, dnaj_3, DnaJ domain signature	C0NFZ6	G3DSA:1.10.287.110, SSF47144, PF07743, SSF46565, PTHR14021, TIGR00714, G3DSA:1.20.1280.20	IPR036869, IPR036386, IPR009073, IPR004640	DnaJ domain, HSC20 (HSCB), C-terminal oligomerisation domain, HSCB C-terminal oligomerisation domain, Chaperone J-domain, IRON-SULFUR CLUSTER CO-CHAPERONE PROTEIN HSCB, Fe-S

							protein assembly co-chaperone HscB, -
LtaPh_3329200	SSF46565, G3DSA:1.10.287.110, PTHR43908, cd06257, PTHR24074, SM00271, PF00226, PR00625	IPR036869, IPR051100, IPR001623, IPR050817	Chaperone J-domain, DnaJ domain, AT29763P-RELATED, DnaJ, CO-CHAPERONE PROTEIN DJLA, dnaj_3, DnaJ domain signature	C1H2E3	G3DSA:1.10.287.110, G3DSA:1.20.1280.20, TIGR00714, SSF47144, SSF46565, cd06257, PTHR14021, PF07743	IPR036869, IPR036386, IPR004640, IPR001623, IPR009073	DnaJ domain, -, Fe-S protein assembly co-chaperone HscB, HSC20 (HSCB), C-terminal oligomerisation domain, Chaperone J-domain, DnaJ, IRON-SULFUR CLUSTER CO-CHAPERONE PROTEIN HSCB, HSCB C-terminal oligomerisation domain
LtaPh_3329200	SSF46565, G3DSA:1.10.287.110, PTHR43908, cd06257, PTHR24074, SM00271, PF00226, PR00625	IPR036869, IPR051100, IPR001623, IPR050817	Chaperone J-domain, DnaJ domain, AT29763P-RELATED, DnaJ, CO-CHAPERONE PROTEIN DJLA, dnaj_3, DnaJ domain signature	Q12350	SSF46565, G3DSA:1.10.287.110, SM00271, PTHR24074	IPR036869, IPR001623, IPR050817	Chaperone J-domain, DnaJ domain, dnaj_3, CO-CHAPERONE PROTEIN DJLA
LtaPh_3329200	SSF46565, G3DSA:1.10.287.110, PTHR43908, cd06257, PTHR24074, SM00271, PF00226, PR00625	IPR036869, IPR051100, IPR001623, IPR050817	Chaperone J-domain, DnaJ domain, AT29763P-RELATED, DnaJ, CO-CHAPERONE PROTEIN DJLA, dnaj_3, DnaJ domain signature	Q8IEP3	PF00226, G3DSA:1.10.287.110, SSF46565, SM00271, cd06257	IPR001623, IPR036869	DnaJ domain, Chaperone J-domain, dnaj_3, DnaJ
TcCLB.507711.120	PTHR16288, SSF50978, G3DSA:2.130.10.10, SSF50998	IPR028884, IPR036322, IPR015943, IPR011047	WD40 REPEAT PROTEIN 4, WD40 repeat-like, -, Quinoprotein alcohol dehydrogenase-like	A4IGH4	SSF50978, PTHR16288, G3DSA:2.130.10.10	IPR036322, IPR028884, IPR015943	WD40 repeat-like, WD40 REPEAT PROTEIN 4, -

TcCLB.507711.120	PTHR16288, SSF50978, G3DSA:2.130.10.10, SSF50998	IPR028884, IPR036322, IPR015943, IPR011047	WD40 REPEAT PROTEIN 4, WD40 repeat-like, -, Quinoprotein alcohol dehydrogenase-like	O74863	PTHR16288, G3DSA:2.130.10.10, SSF50978	IPR028884, IPR015943, IPR036322	WD40 REPEAT PROTEIN 4, -, WD40 repeat-like
TcCLB.507711.120	PTHR16288, SSF50978, G3DSA:2.130.10.10, SSF50998	IPR028884, IPR036322, IPR015943, IPR011047	WD40 REPEAT PROTEIN 4, WD40 repeat-like, -, Quinoprotein alcohol dehydrogenase-like	Q03774	SSF50978, PTHR16288, G3DSA:2.130.10.10	IPR036322, IPR028884, IPR015943	WD40 repeat-like, WD40 REPEAT PROTEIN 4, -
TcCLB.507711.120	PTHR16288, SSF50978, G3DSA:2.130.10.10, SSF50998	IPR028884, IPR036322, IPR015943, IPR011047	WD40 REPEAT PROTEIN 4, WD40 repeat-like, -, Quinoprotein alcohol dehydrogenase-like	Q94GP6	G3DSA:2.130.10.10, SSF50978, PTHR16288	IPR015943, IPR036322, IPR028884	-, WD40 repeat-like, WD40 REPEAT PROTEIN 4
TcCLB.507711.120	PTHR16288, SSF50978, G3DSA:2.130.10.10, SSF50998	IPR028884, IPR036322, IPR015943, IPR011047	WD40 REPEAT PROTEIN 4, WD40 repeat-like, -, Quinoprotein alcohol dehydrogenase-like	Q9EP82	SSF50978, PTHR16288, G3DSA:2.130.10.10	IPR036322, IPR028884, IPR015943	WD40 repeat-like, WD40 REPEAT PROTEIN 4, -
TcIL3000_10_10140	PTHR16288, SSF50978, G3DSA:2.130.10.10	IPR028884, IPR036322, IPR015943	WD40 REPEAT PROTEIN 4, WD40 repeat-like, -	Q5AH60	G3DSA:2.130.10.10, SSF50978, PTHR16288	IPR015943, IPR036322, IPR028884	-, WD40 repeat-like, WD40 REPEAT PROTEIN 4
LINF_120016700	PTHR11533, cd09601, G3DSA:1.10.390.10, PF01433, G3DSA:2.60.40.1730, SSF63737, PF17900	IPR050344, IPR034016, IPR027268, IPR014782, IPR042097, IPR045357	PROTEASE M1 ZINC METALLOPROTEASE, M1_APN-Q_like, Neutral Protease Domain 2, Peptidase family M1 domain, tricorn interacting facor f3 domain, Leukotriene A4 hydrolase N-terminal domain, Peptidase M1 N-terminal domain	A0A0D2DTU3	G3DSA:2.60.40.1730, PTHR15137, G3DSA:1.10.390.10, SSF63737	IPR042097, IPR037813, IPR027268	tricorn interacting facor f3 domain, TRANSCRIPTION INITIATION FACTOR TFIID, Neutral Protease Domain 2, Leukotriene A4 hydrolase N-terminal domain

LINF_120016700	PTHR11533, cd09601, G3DSA:1.10.390.10, PF01433, G3DSA:2.60.40.1730, SSF63737, PF17900	IPR050344, IPR034016, IPR027268, IPR014782, IPR042097, IPR045357	PROTEASE M1 ZINC METALLOPROTEASE, M1_APN-Q_like, Neutral Protease Domain 2, Peptidase family M1 domain, tricorn interacting facor f3 domain, Leukotriene A4 hydrolase N-terminal domain, Peptidase M1 N-terminal domain	A0A1C1CSD0	G3DSA:2.60.40.1730, SSF63737, G3DSA:1.10.390.10, PTHR15137	IPR042097, IPR027268, IPR037813	tricorn interacting facor f3 domain, Leukotriene A4 hydrolase N-terminal domain, Neutral Protease Domain 2, TRANSCRIPTION INITIATION FACTOR TFIID
LINF_120016700	PTHR11533, cd09601, G3DSA:1.10.390.10, PF01433, G3DSA:2.60.40.1730, SSF63737, PF17900	IPR050344, IPR034016, IPR027268, IPR014782, IPR042097, IPR045357	PROTEASE M1 ZINC METALLOPROTEASE, M1_APN-Q_like, Neutral Protease Domain 2, Peptidase family M1 domain, tricorn interacting facor f3 domain, Leukotriene A4 hydrolase N-terminal domain, Peptidase M1 N-terminal domain	A0A1D8PQF6	G3DSA:1.10.390.10, PTHR15137, SSF63737, G3DSA:2.60.40.1730	IPR027268, IPR037813, IPR042097	Neutral Protease Domain 2, TRANSCRIPTION INITIATION FACTOR TFIID, Leukotriene A4 hydrolase N-terminal domain, tricorn interacting facor f3 domain
LINF_120016700	PTHR11533, cd09601, G3DSA:1.10.390.10, PF01433, G3DSA:2.60.40.1730, SSF63737, PF17900	IPR050344, IPR034016, IPR027268, IPR014782, IPR042097, IPR045357	PROTEASE M1 ZINC METALLOPROTEASE, M1_APN-Q_like, Neutral Protease Domain 2, Peptidase family M1 domain, tricorn interacting facor f3 domain, Leukotriene A4 hydrolase N-terminal	C1H2G2	SSF63737, PTHR15137, G3DSA:2.60.40.1730, G3DSA:1.10.390.10	IPR042097, IPR037813, IPR027268	Leukotriene A4 hydrolase N-terminal domain, TRANSCRIPTION INITIATION FACTOR TFIID, tricorn interacting facor f3 domain, Neutral Protease Domain 2

			domain, Peptidase M1 N-terminal domain				
LINF_120016700	PTHR11533, cd09601, G3DSA:1.10.390.10, PF01433, G3DSA:2.60.40.1730, SSF63737, PF17900	IPR050344, IPR034016, IPR027268, IPR014782, IPR042097, IPR045357	PROTEASE M1 ZINC METALLOPROTEASE, M1_APN-Q_like, Neutral Protease Domain 2, Peptidase family M1 domain, tricorn interacting facor f3 domain, Leukotriene A4 hydrolase N-terminal domain, Peptidase M1 N-terminal domain	F1LNY6	SSF63737, G3DSA:1.10.390.10, SSF48371, PTHR15137, G3DSA:2.60.40.1730	IPR042097, IPR027268, IPR016024, IPR037813	Leukotriene A4 hydrolase N-terminal domain, Neutral Protease Domain 2, ARM repeat, TRANSCRIPTION INITIATION FACTOR TFIID, tricorn interacting facor f3 domain
TcCLB.510187.140	No domain detected	No domain detected	No domain detected	A0A0K0K0X7	PF04190, G3DSA:1.25.40.10, PTHR12875	IPR007317, IPR011990	Golgi to ER traffic protein 4, Tetratricopeptide repeat domain, UNCHARACTERIZED
TcCLB.510187.140	No domain detected	No domain detected	No domain detected	No domain detected	No domain detected	No domain detected	No domain detected
TcCLB.510187.140	No domain detected	No domain detected	No domain detected	Q54TH4	PF04190, PTHR12875, G3DSA:1.25.40.10	IPR007317, IPR011990	Golgi to ER traffic protein 4, UNCHARACTERIZED, Tetratricopeptide repeat domain
TcCLB.510187.140	No domain detected	No domain detected	No domain detected	Q6GKV1	G3DSA:1.25.40.10, PTHR12875, PF04190	IPR011990, IPR007317	Tetratricopeptide repeat domain, UNCHARACTERIZED, Golgi to ER traffic protein 4
TcCLB.510187.140	No domain detected	No domain detected	No domain detected	Q8IL82	G3DSA:1.25.40.10, PTHR12875, PF04190	IPR011990, IPR007317	Tetratricopeptide repeat domain, UNCHARACTERIZED,

							Golgi to ER traffic protein 4
LtaPh_3406000	G3DSA:1.25.40.10, PTHR13768, SSF48452	IPR011990, IPR000744	Tetratricopeptide repeat domain, SOLUBLE NSF ATTACHMENT PROTEIN SNAP, TPR-like	A0A3Q0KFW4	SSF48452, PTHR13768, G3DSA:1.25.40.10	IPR011990, IPR000744	TPR-like, SOLUBLE NSF ATTACHMENT PROTEIN SNAP, Tetratricopeptide repeat domain
LtaPh_3406000	G3DSA:1.25.40.10, PTHR13768, SSF48452	IPR011990, IPR000744	Tetratricopeptide repeat domain, SOLUBLE NSF ATTACHMENT PROTEIN SNAP, TPR-like	C0P182	PF04190, PTHR12875, G3DSA:1.25.40.10	IPR007317, IPR011990	Golgi to ER traffic protein 4, UNCHARACTERIZED, Tetratricopeptide repeat domain
LtaPh_3406000	G3DSA:1.25.40.10, PTHR13768, SSF48452	IPR011990, IPR000744	Tetratricopeptide repeat domain, SOLUBLE NSF ATTACHMENT PROTEIN SNAP, TPR-like	C1GXR3	G3DSA:1.25.40.10, PF04190, PTHR12875	IPR011990, IPR007317	Tetratricopeptide repeat domain, Golgi to ER traffic protein 4, UNCHARACTERIZED
LtaPh_3406000	G3DSA:1.25.40.10, PTHR13768, SSF48452	IPR011990, IPR000744	Tetratricopeptide repeat domain, SOLUBLE NSF ATTACHMENT PROTEIN SNAP, TPR-like	U7PSW4	PF04190, G3DSA:1.25.40.10, PTHR12875	IPR007317, IPR011990	Golgi to ER traffic protein 4, Tetratricopeptide repeat domain, UNCHARACTERIZED
LINF_320046700	PTHR19980, G3DSA:1.25.40.10, SSF48452	IPR045243, IPR011990	RNA CLEAVAGE STIMULATION FACTOR, Tetratricopeptide repeat domain, TPR-like	A0A158Q688	SSF48452, PF05843, PTHR19980	IPR011990, IPR008847, IPR045243	TPR-like, Suppressor of forked protein (Suf), RNA CLEAVAGE STIMULATION FACTOR
LINF_320046700	PTHR19980, G3DSA:1.25.40.10, SSF48452	IPR045243, IPR011990	RNA CLEAVAGE STIMULATION FACTOR, Tetratricopeptide repeat domain, TPR-like	F1QIB2	PTHR19980, SM00386, PF05843, SSF48452	IPR045243, IPR003107, IPR008847, IPR011990	RNA CLEAVAGE STIMULATION FACTOR, hat_new_1, Suppressor of forked protein (Suf), TPR-like

LINF_320046700	PTHR19980, G3DSA:1.25.40.10, SSF48452	IPR045243, IPR011990	RNA CLEAVAGE STIMULATION FACTOR, Tetratricopeptide repeat domain, TPR-like	Q12996	PTHR19980, PF05843, SSF48452, SM00386	IPR045243, IPR008847, IPR011990, IPR003107	RNA CLEAVAGE STIMULATION FACTOR, Suppressor of forked protein (Suf), TPR-like, hat_new_1
LINF_320046700	PTHR19980, G3DSA:1.25.40.10, SSF48452	IPR045243, IPR011990	RNA CLEAVAGE STIMULATION FACTOR, Tetratricopeptide repeat domain, TPR-like	Q19866	SSF48452, PF05843, PTHR19980	IPR011990, IPR008847, IPR045243	TPR-like, Suppressor of forked protein (Suf), RNA CLEAVAGE STIMULATION FACTOR
LINF_320046700	PTHR19980, G3DSA:1.25.40.10, SSF48452	IPR045243, IPR011990	RNA CLEAVAGE STIMULATION FACTOR, Tetratricopeptide repeat domain, TPR-like	Q99LI7	SM00386, SSF48452, PF05843, PTHR19980	IPR003107, IPR011990, IPR008847, IPR045243	hat_new_1, TPR-like, Suppressor of forked protein (Suf), RNA CLEAVAGE STIMULATION FACTOR
LINF_290018900	No domain detected	No domain detected	No domain detected	I1MDZ1	PTHR12184, PF03981	IPR007129, IPR021150	UBIQUINOL-CYTOCHROME C REDUCTASE COMPLEX ASSEMBLY FACTOR 1 FAMILY MEMBER, Ubiquinol-cytochrome C chaperone
LINF_290018900	No domain detected	No domain detected	No domain detected	P21560	PTHR12184, PF03981	IPR007129, IPR021150	UBIQUINOL-CYTOCHROME C REDUCTASE COMPLEX ASSEMBLY FACTOR 1 FAMILY MEMBER, Ubiquinol-cytochrome C chaperone
LINF_290018900	No domain detected	No domain detected	No domain detected	Q5AC35	PTHR12184, PF03981	IPR007129, IPR021150	UBIQUINOL-CYTOCHROME C REDUCTASE COMPLEX

							ASSEMBLY FACTOR 1 FAMILY MEMBER, Ubiquinol-cytochrome C chaperone
LINF_290018900	No domain detected	No domain detected	No domain detected	Q7XHR1	PF03981, PTHR12184	IPR021150, IPR007129	Ubiquinol-cytochrome C chaperone, UBIQUINOL-CYTOCHROME C REDUCTASE COMPLEX ASSEMBLY FACTOR 1 FAMILY MEMBER
LINF_290018900	No domain detected	No domain detected	No domain detected	Q8I661	PTHR12184, PF03981	IPR007129, IPR021150	UBIQUINOL-CYTOCHROME C REDUCTASE COMPLEX ASSEMBLY FACTOR 1 FAMILY MEMBER, Ubiquinol-cytochrome C chaperone
Lsey_0010_0360	No domain detected	No domain detected	No domain detected	C0NNU2	G3DSA:3.40.50.410, PTHR12831, PF03850	IPR036465, IPR004600	von Willebrand factor, type A domain, TRANSCRIPTION INITIATION FACTOR IIH TFIIH , POLYPEPTIDE 3-RELATED, Transcription factor Tfb4
